# Induction of natural IgE by glucocorticoids

**DOI:** 10.1084/jem.20220903

**Published:** 2022-09-13

**Authors:** Jaechul Lim, Erica V. Lin, Jun Young Hong, Bharat Vaidyanathan, Steven A. Erickson, Charles Annicelli, Ruslan Medzhitov

**Affiliations:** 1 Department of Immunobiology, Yale University School of Medicine, New Haven, CT; 2 Department of Systems Biology, College of Life Science and Biotechnology, Yonsei University, Seoul, South Korea; 3 Howard Hughes Medical Institute, Yale University School of Medicine, New Haven, CT

## Abstract

IgE mediates allergic responses by coating mast cell or basophil surfaces and inducing degranulation upon binding a specific allergen. IgE can also be spontaneously produced in the absence of foreign allergens; yet the origin, regulation, and functions of such “natural” IgE still remain largely unknown. Here, we find that glucocorticoids enhance the production of IgE in B cells both in vivo and ex vivo without antigenic challenge. Such IgE production is promoted by B cell–intrinsic glucocorticoid receptor signaling that reinforces CD40 signaling and synergizes with the IL-4/STAT6 pathway. In addition, we found that rare B cells in the mesenteric lymph nodes are responsible for the production of glucocorticoid-inducible IgE. Furthermore, locally produced glucocorticoids in the gut may induce natural IgE during perturbations of gut homeostasis, such as dysbiosis. Notably, mice preemptively treated with glucocorticoids were protected from subsequent pathogenic anaphylaxis. Together, our results suggest that glucocorticoids, classically considered to be broadly immunosuppressive, have a selective immunostimulatory role in B cells.

## Teaser

The present study reveals that glucocorticoids promote the production of IgE by rare B cells in the mesenteric lymph nodes in the absence of experimentally administered antigens.

## Graphical Abstract

**Figure:**
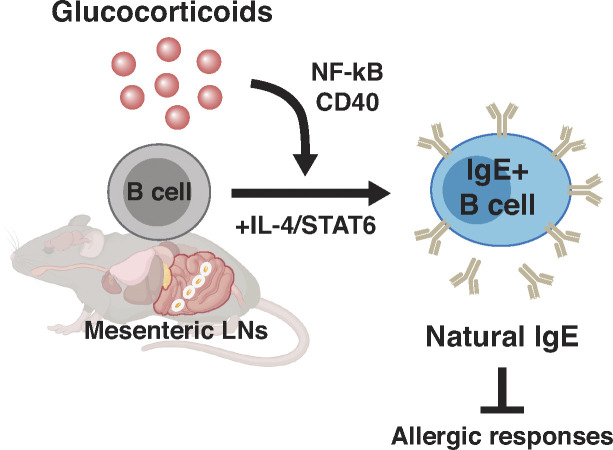

## Introduction

Allergy, a leading chronic condition affecting hundreds of millions of people worldwide, targets diverse and seemingly innocuous environmental stimuli such as pollen or peanut by activating type 2 immune responses (Lloyd and Snelgrove, 2018; Palm et al., 2012). Unlike type 1 immunity, which deals with pathogens such as bacteria and viruses, type 2 immunity defends the body from macroparasites, noxious chemicals, and toxins (Iwasaki and Medzhitov, 2015; Palm et al., 2012). Type 2 immunity is mediated primarily by T helper 2 cells alongside B cells that secrete IgE and IgG1 antibodies (Lloyd and Snelgrove, 2018; Palm et al., 2012). In the initial presence of allergen and T cell signals, allergen-specific B cells undergo class switch recombination and somatic hypermutation (SHM) to produce high-affinity IgE, which coats the high-affinity Fc epsilon receptor I (FcεRI) expressed on surfaces of innate immune sentinels like mast cells and basophils (He et al., 2015; Oettgen, 2016; Stavnezer and Schrader, 2014). While IgE-mediated inflammatory effector responses, such as extravasation, mucus secretion, and gut peristalsis, are considered protective against helminths and noxious chemicals, they can also be pathological if misdirected or excessive (Anthony et al., 2007; Palm et al., 2012). Indeed, when bound to typically nonharmful allergens, allergen-specific IgE can crosslink FcεRI receptors and prompt mast cells and basophils to release inflammatory mediators like histamine, thus eliciting downstream symptoms ranging from a mild itch to anaphylactic shock that is associated with life-threatening hypotension and airway closure (Galli and Tsai, 2012; Kelly and Grayson, 2016; Reber et al., 2017).

Curiously, IgE can also be spontaneously induced even in the absence of specific environmental allergens or microbiota- or food-driven antigens and independently of antigen presentation or T cell costimulation (McCoy et al., 2006; Pabst and Slack, 2020). Such IgE antibodies have been termed “natural” IgE, after natural IgM antibodies of similar broad specificity (McCoy et al., 2006), and may play homeostatic roles in cancer surveillance or skin barrier defenses (Crawford et al., 2018; Hayes et al., 2020). The spontaneous induction and homeostatic capacities of natural IgE thus suggest that physiological or environmental signals may be sufficient to induce basal levels of natural IgE with potentially protective roles. For example, germ-free mice display increased basal IgE levels and food antigen–dependent gut T helper 2 cell responses (Hong et al., 2019; Kim et al., 2016), as well as increased expression in the mesenteric LNs (mLNs) of epsilon germline transcripts (εGLTs) required for IgE switching (Cahenzli et al., 2013; Hill et al., 2012). Furthermore, gut dysbiosis induced by broad-spectrum antibiotics or genetic defects in toll-like receptor signaling is associated with increased inflammation as well as elevated IgE levels, suggesting that perturbed microbial signals coupled with inflammatory signals may facilitate IgE induction (Gavin et al., 2006; Hill et al., 2012; Schnare et al., 2001; Wesemann and Nagler, 2016). However, it remains unclear how such broadly specific or nonspecific natural IgE is produced and what functions it may hold (Kelly and Grayson, 2016).

Glucocorticoids are endogenous steroid hormone signals implicated in the general stress response and used as immunosuppressive drugs for treating allergies (Cain and Cidlowski, 2017). However, recent literature posits that glucocorticoids have cell type–specific and dose-dependent effects on immune responses, which align with their multiple modes of action (Franco et al., 2019; Sasse et al., 2019; Weikum et al., 2017). In B cells, glucocorticoids have been shown to alter B cell maturity and lifespan, which can dictate downstream antibody production or affinity to certain antigens (Cain et al., 2020; Gruver-Yates et al., 2014). Corticosteroids have also been implicated in the allergic response by altering type 2 cytokine production in human and murine T cells (Blotta et al., 1997; Ohnishi et al., 2008), and by increasing or decreasing IgE levels in human peripheral blood mononuclear cells (PBMCs) by regulating CD40L-CD40 (Jabara et al., 2001; Salvi et al., 2000). Due to pleiotropic effects, however, detailed molecular mechanisms and functions of glucocorticoids in allergy have been debated and remain poorly understood to date (Lane and Kemeny, 1994; Pullerits et al., 2002).

Here, we show that glucocorticoids can act directly on B cells to promote the synthesis of “natural” IgE with potential protective functions in allergy. We define “natural” IgE as IgE induced in the absence of experimentally administered antigen. Using an ex vivo murine B cell culture, we found that glucocorticoids induce B cells to undergo class switch recombination to IgE by reinforcing and substituting for CD40 signaling in a B cell–intrinsic and IL-4/STAT6–dependent manner. We also linked glucocorticoids to elevated basal natural IgE in the absence of experimentally added antigen in vivo, which was secreted locally by rare B cells with a plasmablast-like signature in the mLNs. Notably, natural IgE induced by glucocorticoids blunted the magnitude of subsequent anaphylactic responses. In all, our work establishes a connection between glucocorticoids and natural IgE that can clarify the potential properties of natural IgE and its B cell producers during allergy.

## Results

### Glucocorticoids increase IgE switching in B cells ex vivo

**Figure 1.**
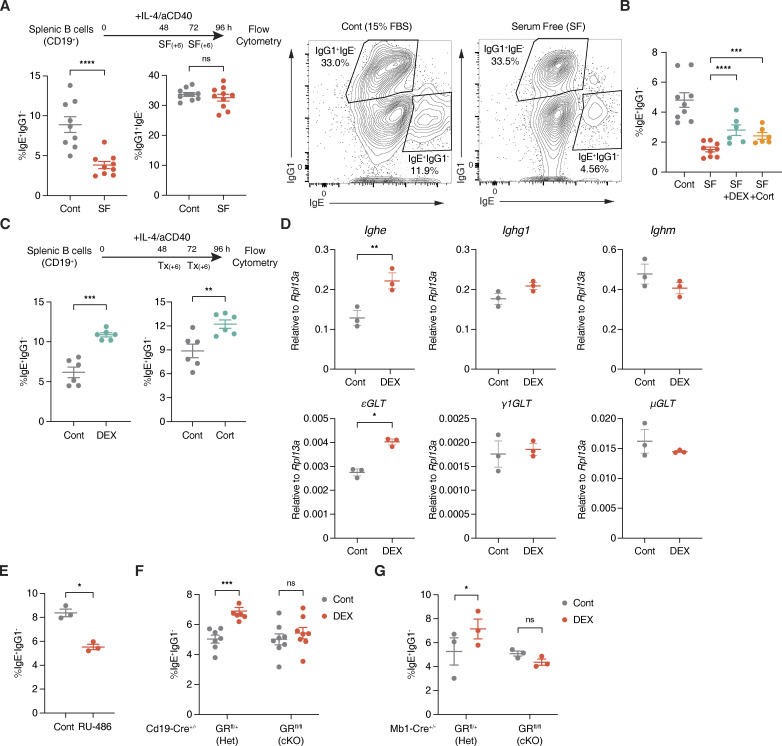
**Glucocorticoids induce IgE switching in B cells. (A)** Experimental setup and representative plots for assessing serum starvation effects on ex vivo B cell class switching. CD19^+^ splenic B cells were cultured with IL-4 and aCD40 in control complete media (Cont, gray) or with SF conditions (red). Numbers indicate the percentage of IgE^+^ IgG1^−^ or IgG1^+^ IgE^−^ B cells within a live B220^+^ population, determined by flow cytometry (*n* = 11–12 mice/group, compiled from three independent experiments). **(B)** Percentage of IgE^+^ IgG1^−^ B cells (within live B220^+^) from B cells cultured in Cont media (gray), SF conditions (red), SF conditions with DEX (teal), or SF conditions with Cort (orange), as described in A (*n* = 6–9 mice/group, compiled from three independent experiments). **(C)** Experimental setup and representative plots for assessing glucocorticoid effects on ex vivo B cell switching. B cells cultured in Cont media were treated in two 6-h windows with vehicle control (Cont, gray) or with DEX or Cort (teal). Numbers indicate the percentage of IgE^+^ IgG1^−^ B cells within a live B220^+^ population by flow cytometry (*n* = 6 mice/group, compiled from two and representative of five independent experiments). **(D)** Relative RT-qPCR expression of mature *Ighe*, *Ighg1*, or *Ighm* transcripts (top) and upstream εGLTs, γ1GLTs, or μGLTs (bottom) for B cells cultured with Cont (gray) or DEX (red). Expression at 96 h was normalized by *Rpl13a* (*n* = 3 mice/group). **(E)** The percentage of IgE^+^ IgG1^−^ B cells (within live B220^+^) from B cells cultured with Cont (gray) or RU-486 (red), as described in C (*n* = 3 mice/group, representative of two independent experiments). **(F)** Percentage of IgE^+^ IgG1^−^ B cells (within live B220^+^) from Cont (gray) or DEX-treated (red) *Cd19-Cre*^+/−^ GR Het vs. cKO B cells, as described in C (*n* = 6–7 mice/group, compiled from three independent experiments). **(G)** Percentage of IgE^+^ IgG1^−^ B cells (within live B220^+^) from Cont (gray) or DEX-treated (red) *Mb1-Cre*^+/−^ GR Het vs. cKO B cells (*n* = 3 mice/group). Error bars in A–G represent SEM. P values <0.05 were considered significant (*, P < 0.05; **, P < 0.01; ***, P < 0.001; ****, P < 0.0001; ns [or unlabeled], not significant). Ratio paired Student’s *t* test was used for A–G.

**Figure S1.**
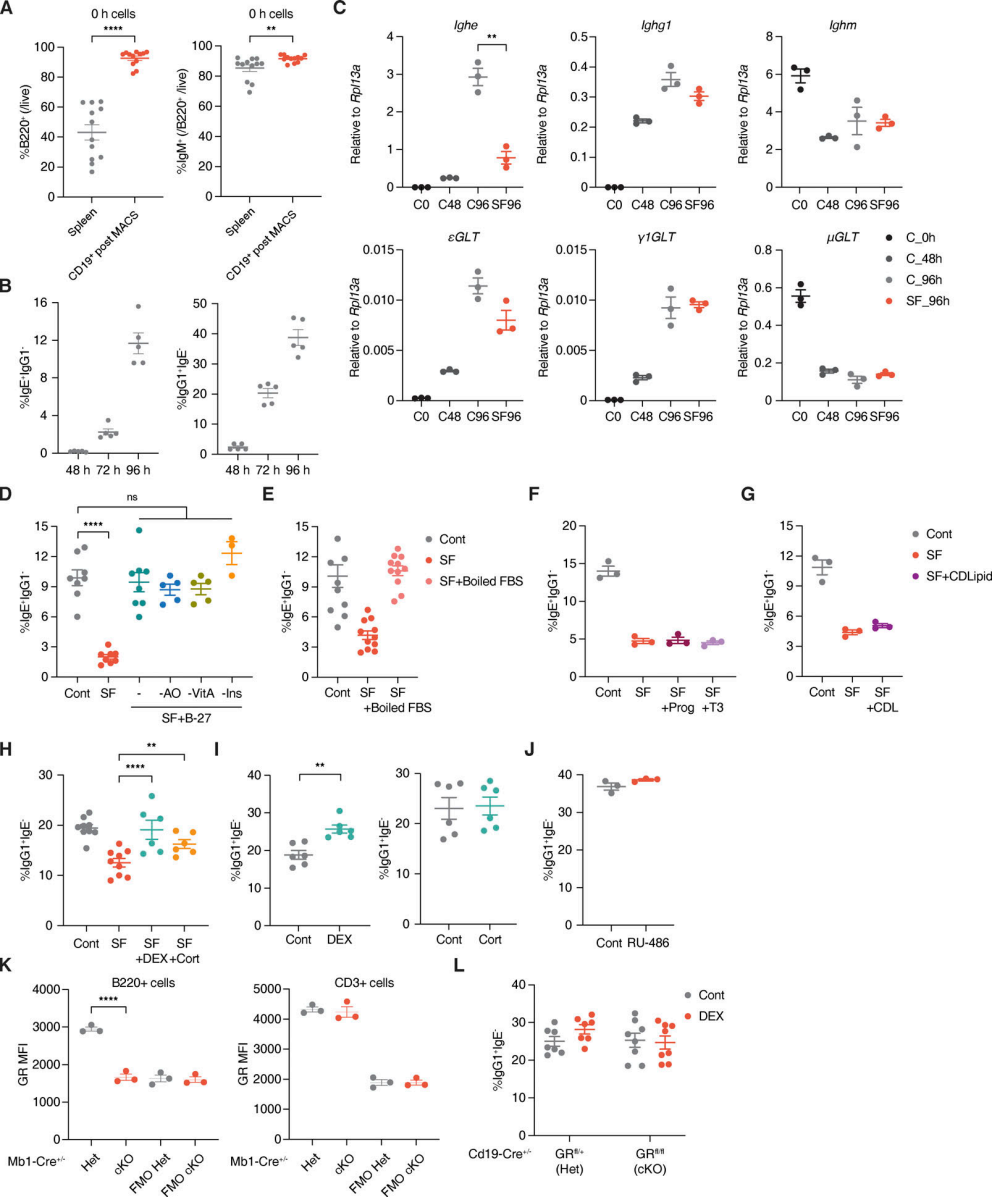
**IgG1 switching is nearly unaffected by glucocorticoids. (A)** Percentage of B220^+^ B cells or IgM^+^ B cells prior to culture (0 h; *n* = 12 mice/group, compiled from four independent experiments). **(B)** Percentage of IgE^+^ IgG1^−^ or IgG1^+^ IgE^−^ B cells (within live B220^+^) from B cells cultured ex vivo for 48, 72, or 96 h (*n* = 5 mice/group, compiled from two independent experiments). **(C)** Relative RT-qPCR expression of mature *Ighe*, *Ighg1*, or *Ighm* transcripts (top) and upstream εGLTs, γ1GLTs, or μGLTs (bottom) for B cells preculture (C0, black), after 48 or 96 h in Cont media (C48, dark gray, or C96, light gray), or after 96 h with SF conditions in two 6-h windows (SF96, red), as similarly described in Fig. 1 C. Expression was normalized by *Rpl13a* (*n* = 3 mice/group). **(D)** Percentage of IgE^+^ IgG1^−^ B cells (within live B220^+^) from B cells cultured in Cont media (gray), SF conditions (red), SF conditions with B-27 (teal), or SF conditions with B-27 variants lacking antioxidants (AO, blue), vitamin A (VitA, green), or insulin (Ins, orange; *n* = 6–8 mice/group, compiled from three independent experiments). **(E)** Percentage of IgE^+^ IgG1^−^ B cells (within live B220^+^) from B cells cultured in Cont media (gray), SF conditions (red), or SF conditions with boiled FBS (pink; *n* = 10–11 mice/group, compiled from four independent experiments). **(F)** Percentage of IgE^+^ IgG1^−^ B cells (within live B220^+^) from B cells cultured in Cont media (gray), SF conditions (red), SF conditions with progesterone (Prog, dark purple), or SF conditions with T3 (light purple; *n* = 3 mice/group). **(G)** Percentage of IgE^+^ IgG1^−^ B cells (within live B220^+^) from B cells cultured in Cont media (gray), SF conditions (red), or SF conditions with chemically defined lipids (CDlipid or CDL, purple; *n* = 3 mice/group). **(H)** Percentage of IgG1^+^ IgE^−^ B cells (within live B220^+^) from B cells cultured in Cont media (gray), SF conditions (red), SF conditions with DEX (teal), or SF conditions with Cort (orange) (*n* = 6–9 mice/group, compiled from three independent experiments). **(I)** Percentage of IgG1^+^ IgE^−^ B cells (within live B220^+^) from B cells cultured in complete media with Cont (gray) or with DEX or Cort (teal, left and right, respectively; *n* = 6 mice/group, compiled from two independent experiments). **(J)** Percentage of IgG1^+^ IgE^-^ B cells (within live B220^+^) from B cells cultured with Cont vehicle (gray) or RU-486 (red; *n* = 3 mice/group, representative of two independent experiments). **(K)** GR expression for live B220^+^ B cells or live CD3^+^ T cells in *Mb1-Cre*^+/−^ GR Het (gray) vs. cKO (red) mice. Numbers indicate GR MFI from stained PBMCs or their FMO controls by flow cytometry (*n* = 3 mice/group). **(L)** Percentage of IgG1^+^ IgE^−^ B cells (within live B220^+^) from Cont (gray) or DEX-treated (red) *Cd19-Cre*^+/−^ GR Het vs. cKO B cells (*n* = 6–7 mice/group, compiled from three independent experiments). Error bars in A–L represent SEM. P values <0.05 were considered significant (**, P < 0.01; ****, P < 0.0001; ns, not significant). Ratio paired Student’s *t* test was used for A–J and L, and one-way ANOVA with post hoc Tukey’s multiple comparisons test was used for K.

While the existence of natural IgE has been established (McCoy et al., 2006), an endogenous signal that induces natural IgE production has not yet been identified, particularly in the absence of any antigenic challenges. We asked whether there is any endogenous signaling molecule present in normal serum that can affect IgE production. To this end, we performed an experiment using ex vivo cultures to screen for efficacy of IgE class switch recombination, whereby mature naive splenic B cells were stimulated by IL-4 and CD40L, which lead to IgE switching (Fig. 1 A and Fig. S1, A and B; Geha et al., 2003). We discovered that transient serum starvation decreased class switching to IgE even in the presence of IL-4 and CD40L stimuli (Fig. 1 A and Fig. S1 C). However, IgG1 class switching was nearly unaffected under the same conditions, suggesting that IgE reduction is not attributable to a general defect in class switch recombination. As normal FBS possesses an uncharacterized composition of diverse factors, it has been challenging to pinpoint specific factors in serum that drive IgE class switch recombination (van der Valk, 2022). To identify specific factors whose absence during serum starvation is responsible for the reduction of IgE^+^ B cells, we thus first utilized B-27, a chemically defined alternative of serum for culturing neurons and stem cells, to circumvent the undefined composition of FBS (Brewer et al., 1993). Interestingly, the addition of B-27 completely rescued the reduction of IgE switching in the serum-free (SF) culture condition, suggesting that B-27 contains one or more factors responsible for IgE switching (Fig. S1 D). To narrow down candidates, we supplemented SF cultures with B-27 variants that lack a subset of components such as antioxidants, vitamin A, or insulin, but none of these defined variants showed a defect in IgE switching (Fig. S1 D). Furthermore, boiled FBS did not reduce B cell switching to IgE, suggesting that protein components in the serum are not responsible for IgE switching (Fig. S1 E). To test the remaining nonprotein components of B-27, we also added chemically defined lipids, progesterone, or the thyroid hormone (T3) to the SF condition, but did not observe any significant restoration of IgE switching (Fig. S1, F and G; and Table S1).

Interestingly, we found that glucocorticoids, steroid stress hormones also present in B-27 and FBS, can contribute to IgE switching. Both corticosterone (Cort) and dexamethasone (DEX), a synthetic glucocorticoid with a higher affinity for the glucocorticoid receptor (GR), significantly restored the fraction of IgE^+^ B cells in the SF condition (Fig. 1 B and Fig. S1 H). Moreover, the addition of Cort or DEX in the control complete media further increased IgE switching (Fig. 1 C). In this condition, IgG1^+^ B cells were also slightly increased by DEX (Fig. S1 I). However, we confirmed that εGLTs required for IgE switching were selectively increased by such DEX treatment, suggestive of direct IgE switching from IgM to IgE at least in our ex vivo setting (Fig. 1 D). Consistent with these observations, incubating B cells cultured in control complete media with RU-486, a GR inhibitor, significantly impaired IgE but not IgG1 class switching (Fig. 1 E and Fig. S1 J). Furthermore, the addition of DEX to complete media cultures of B cells from mice with B cell–specific deletions of the *Nr3c1* GR gene (cKO, either by *Cd19-Cre* or *Mb1-Cre*) did not increase IgE class switching (Fig. 1, F and G; and Fig. S1, K and L). Together, our data thus suggest that glucocorticoids induce B cell class switching to IgE ex vivo in a B cell–intrinsic manner.

### Glucocorticoids reinforce CD40 signaling and plasmablast-like gene expression

**Figure 2.**
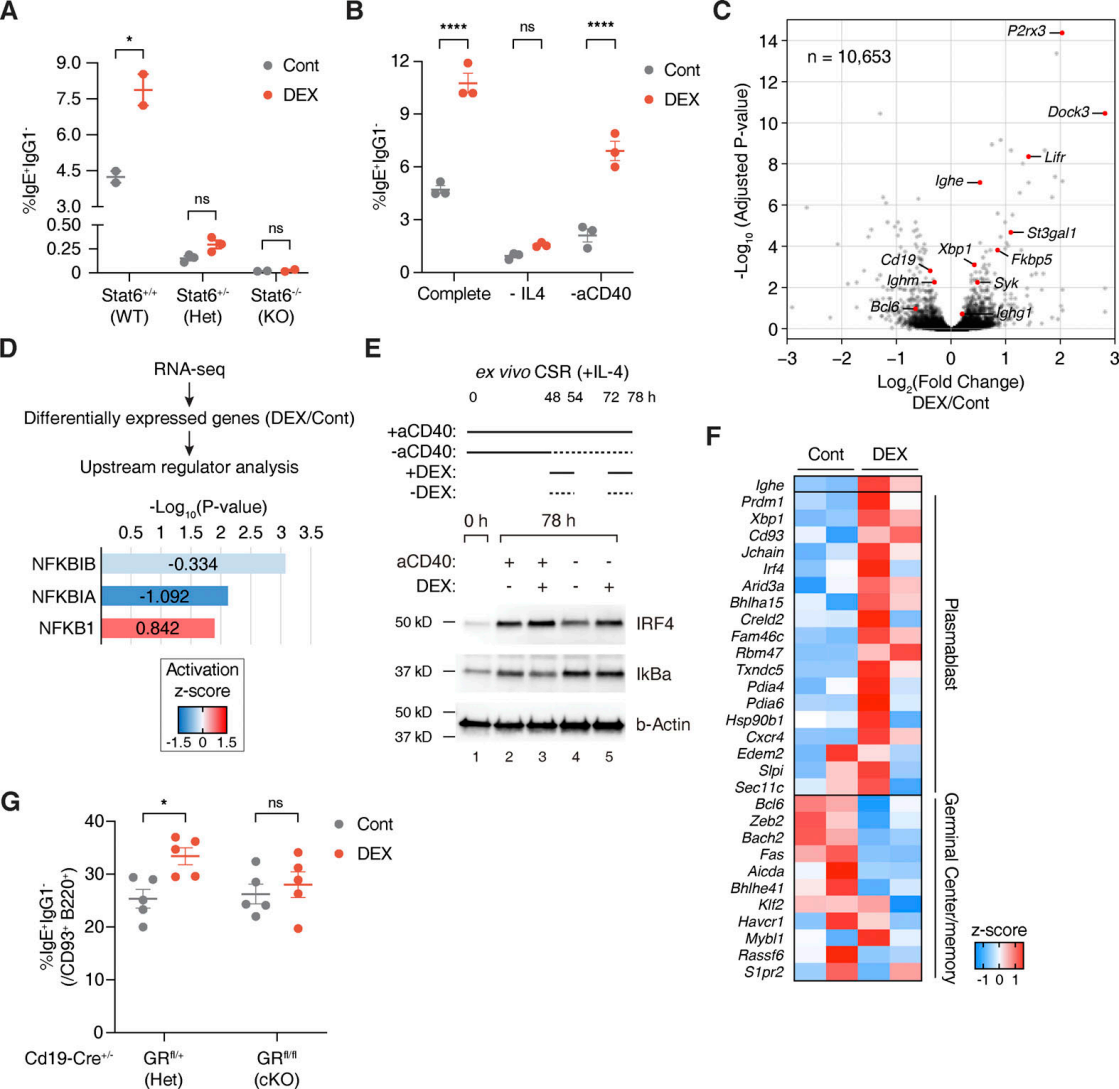
**Glucocorticoids promote CD40 signaling and plasmablast-like gene expression ex vivo. (A)** Percentage of IgE^+^ IgG1^−^ B cells (within live B220^+^) from CD19^+^ splenic B cells of *Stat6* WT, Het, or KO mice cultured with Cont (gray) or DEX (red), as described in Fig. 1 C (*n* = 2–3 mice/group, representative of two independent experiments). **(B)** Percentage of IgE^+^ IgG1^−^ B cells (within live B220^+^) from WT B cells subjected to IL-4 or aCD40 depletion and treated with Cont (gray) or DEX (red). “Complete” media contained IL-4 and aCD40 (*n* = 3 mice/group, representative of two independent experiments). **(C)** Volcano plot showing differentially expressed genes between B cells cultured ex vivo with DEX or Cont. x axis indicates log_2_(fold change) of counts per million (CPM) and y axis indicates corresponding −log_10_(adjusted P value) for differential expression, with key B cell or differentially expressed genes highlighted in red (*n* = 2 mice/group as biological replicates). **(D)** Effect of DEX on B cell activation of upstream regulators NFKB1 (p105), NFKBIA (IκBa), and NFKBIB (IκBb). WT Cont or DEX-treated B cells were analyzed by RNA-seq and pathway analysis for upstream regulators of differentially expressed targets. x axis indicates −log_10_(P value) for overlap between DEX-induced changes in upstream regulator targets and observed gene expression changes (relative to Cont), by Fisher’s exact test. Numbers and color gradations indicate activation z-score (*n* = 2 mice/group as biological replicates). **(E)** Effect of DEX treatment during aCD40 depletion on IRF4 and IκBa protein abundance by Western blotting. WT B cells were harvested preculture (naive B cells) or after 78 h of complete (lanes 2–3) or aCD40-depleted (lanes 4–5) cultures with Cont or Dex treatment, as similarly described in B (representative of two biological replicates). **(F)** Heat map showing relative expression of genes associated with plasmablast-like or germinal center and memory B cell–like signatures for Cont vs. DEX-treated IgE^+^ B cells sorted after culture. Color gradations indicate *z*-scores by CPM (*n* = 2 mice/group as biological replicates). **(G)** Percentage of IgE^+^ IgG1^−^ cells (within live B220^+^ CD93^+^) from *Cd19-Cre*^+/−^ GR Het vs. cKO B cells cultured with Cont (gray) or DEX (red; *n* = 5 mice/group, compiled from two independent experiments). Error bars in A–B and G represent SEM. P values <0.05 were considered significant (*, P < 0.05; ****, P < 0.0001; ns, not significant). Ratio-paired Student’s *t* test was used for A–B and G. Source data are available for this figure: SourceData F2.

**Figure S2.**
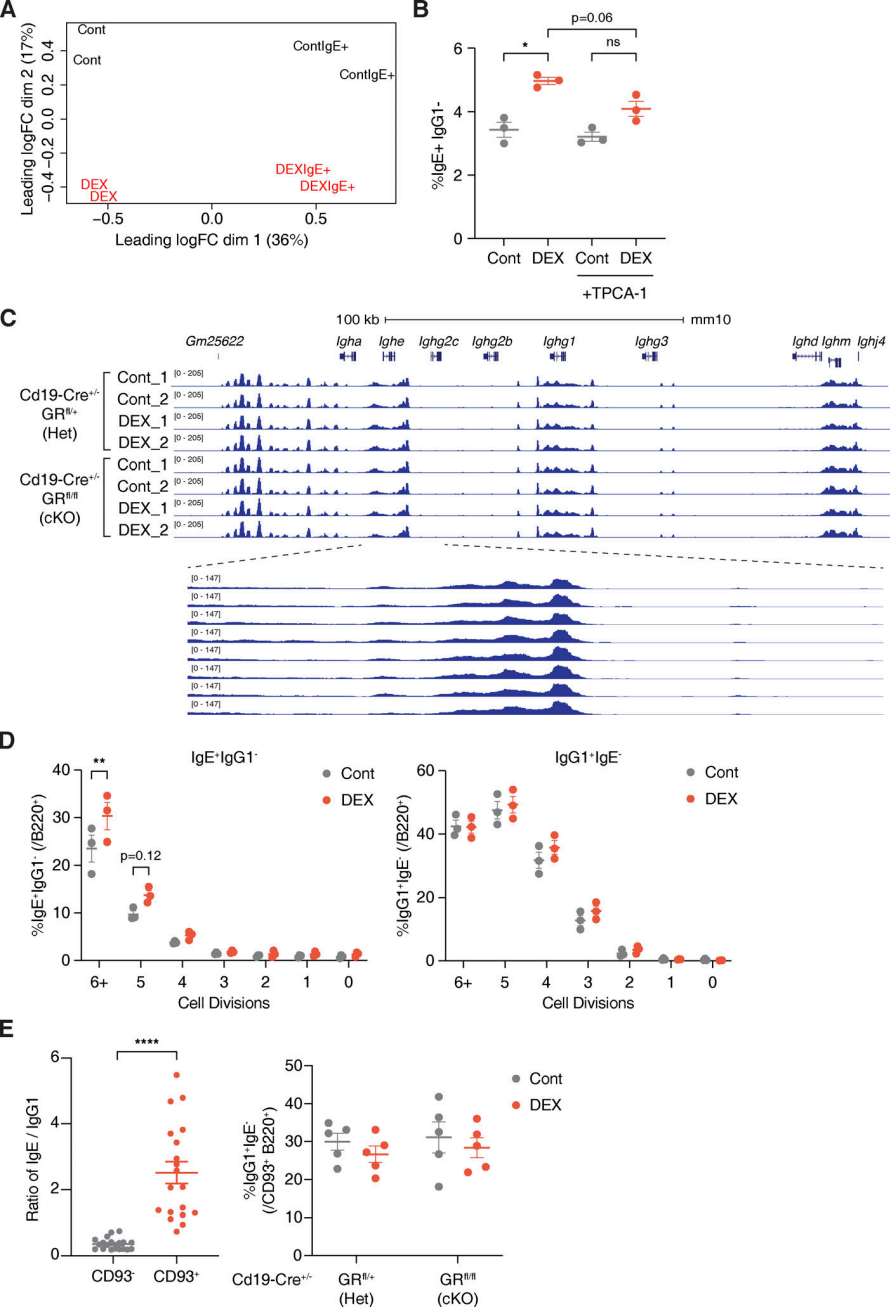
**Glucocorticoids support the activation of IgE**^**+**^ **B cells and promote IgE production without global changes in chromatin accessibility. (A)** Multidimensional scaling plot representing relative positions for Cont vs. DEX-treated total B cells (Cont and DEX) or IgE^+^ B cells sorted postculture (ContIgE^+^ and DEXIgE^+^). x and y axes represent leading log_2_(CPM fold change) for DEX/Cont in the first and second dimension, respectively (*n* = 2 mice/group as biological replicates). **(B)** Percentage of IgE^+^ IgG1^−^ B cells (within live B220^+^) from B cells cultured with Cont vehicle (gray) or DEX (red) without or with TPCA-1 (*n* = 3 mice/group). **(C)** Chromatin accessibility within the *IgH* locus of *Cd19-Cre*^+/−^ GR Het vs. cKO B cells upon DEX treatment, determined by ATAC-seq. 100 kb scale is shown for *IgH*, with blue boxes representing exons (top). Dashed lines designate a magnified view of *Ighe* (bottom; *n* = 1–3 pooled mice/sample with *n* = 2 samples/group). **(D)** Percentages of IgE^+^ or IgG1^−^ B cells at each cell division within B220^+^ cells after 96 h, determined by CellTrace labeling (*n* = 3 mice/group). **(E)** Ratio of percentage of IgE^+^ to IgG1^+^ B cells within live B220^+^ CD93^−^ cells (gray) or within live B220^+^ CD93^+^ cells (red) from WT Cont B cells (left), and absolute percentage of IgG1^+^ IgE^−^ B cells (within live B220^+^ CD93^+^) from Cont (gray) or DEX-treated (red) *Cd19-Cre*^+/−^ GR Het vs. cKO B cells (right; *n* = 19 mice/group, compiled from six independent experiments [left]; *n* = 5 mice/group, compiled from two independent experiments [right]). Error bars in B, D, and E represent SEM. P values <0.05 were considered significant (*, P < 0.05; **, P < 0.01; ****, P < 0.0001; unlabeled, not significant). Ratio paired Student’s *t* test was used for B and E, and two-way ANOVA with post hoc Šídák’s multiple comparisons test was used for D.

Next, we asked how glucocorticoids induce IgE switching. Mechanistically, activation of the STAT6 transcription factor through IL-4/IL-4R signaling has been described in the literature to be critical for IgE switching (Linehan et al., 1998). DEX failed to induce IgE switching in ex vivo cultures of mature naive splenic B cells from *Stat6* knockout mice, suggesting that glucocorticoid induction of IgE switching depends on STAT6 (Fig. 2 A). In line with this, IgE switching was not induced by DEX in WT B cells that were depleted of IL-4 from the culture media (Fig. 2 B). Interestingly, however, we observed a significant induction of IgE switching by DEX in B cells depleted of the CD40L stimulus, implying that glucocorticoids may compensate for the loss of CD40L, at least ex vivo (Fig. 2 B). To better understand the underlying molecular mechanism, we performed RNA sequencing (RNA-seq) on B cells cultured ex vivo with or without DEX (Fig. 2 C, Fig. S2 A, and Table S2). Supporting the earlier notion, NF-κB, an important target of CD40 signaling in B cells, was significantly activated by DEX in the RNA-seq data (Fig. 2 D; Geha et al., 2003; Luo et al., 2018; Mizuno and Rothstein, 2005). As glucocorticoids are canonically known to inhibit rather than activate NF-κB target transcription (Tan and Wahli, 2016), we validated if NF-κB can indeed be activated by DEX by measuring the protein levels of IκBa, an inhibitor of NF-κB, and IRF4, a downstream target of NF-κB (Grumont and Gerondakis, 2000). Consistent with our sequencing data, the level of IκBa in cultured B cells was reduced by DEX, while the level of IRF4 was increased (Fig. 2 E). Moreover, suppression of NF-κB activation during DEX treatment by using a selective NF-κB inhibitor (TPCA-1, reversible inhibitor of IκB kinase) reversed the effect of DEX (Fig. S2 B). Of note, however, we did not detect changes in the binding of GR to immunoglobulin genes or in chromatin accessibility of the *IgH* locus (Fig. S2 C). Therefore, our data collectively suggest that glucocorticoids promote CD40L/CD40 signaling to NF-κB in B cells to induce downstream IgE class switching.

In addition, we found that highly divided IgE^+^ B cells (>5 divisions) were specifically increased by DEX treatment (Fig. S2 D). As the activation of IgE^+^ B cells is closely linked to cell proliferation (Wu et al., 2017), DEX may support the activation of B cells and promote IgE production. To examine the effect of DEX specifically on IgE^+^ B cells, we sequenced total RNA from IgE^+^ B cells sorted after culture by FACS (Fig. 2 F and Fig. S2 A). As compared to control IgE^+^ B cells, DEX-treated IgE^+^ B cells showed a stronger plasmablast-like signature, including *Prdm1*, *Cd93*, and *Xbp1* (Fig. 2 F and Table S2; Brynjolfsson et al., 2018; Croote et al., 2018; Glaros et al., 2021). Moreover, DEX was able to augment IgE switching in CD93^+^ B cells that can be either differentiating plasmablasts/plasma cells or immature B cells, the latter of which have been previously reported to switch preferentially to IgE over IgG1 through increased direct IgE switching (Fig. 2 G and Fig. S2 E; Wesemann et al., 2011). Thus, our data suggest that glucocorticoids may establish a distinct transcriptome in B cells that skews toward and sets the stage for IgE production by altering B cell maturity and fate.

### Natural IgE is induced in mLNs by glucocorticoids in vivo

**Figure 3.**
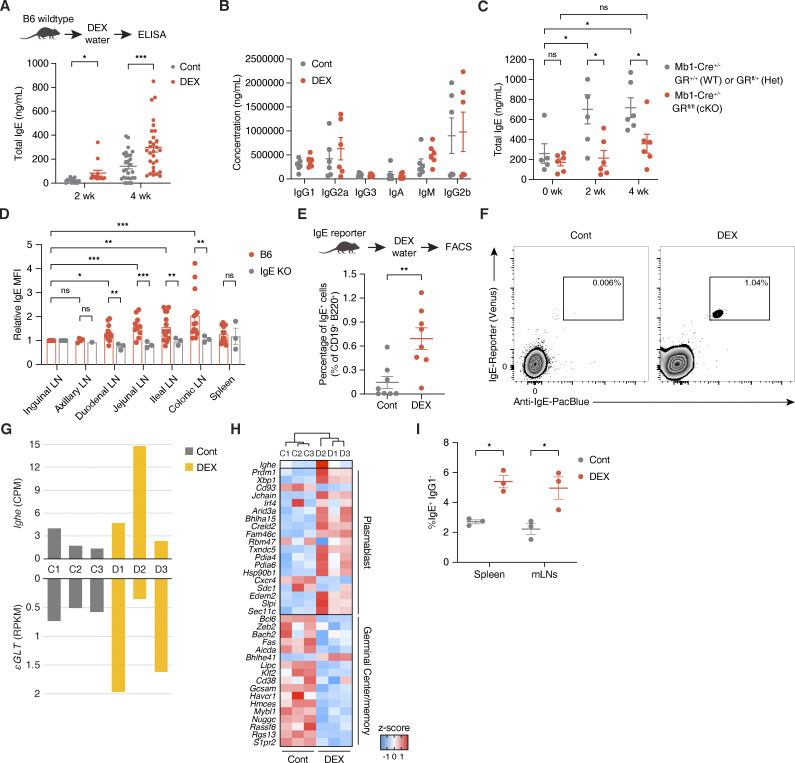
**Natural IgE is produced in mLNs by glucocorticoids in vivo. (A)** Total plasma IgE concentration of WT mice treated with Cont (gray) vs. DEX (red) in drinking water for 2 or 4 wk, as represented in the experimental setup (*n* = 29 mice/group, compiled across five independent experiments). **(B)** Total plasma IgG1, IgG2a, IgG3, IgA, IgM, or IgG2b concentration of WT mice after 4 wk of Cont (gray) vs. DEX (red) treatment (*n* = 6 mice/group, compiled across two independent experiments). **(C)** Total plasma IgE concentration of *Mb1-Cre*^+/−^ GR WT or Het (gray) vs. cKO (red) mice at steady state (0 wk), or after 2 or 4 wk of DEX treatment (*n* = 5–6 mice/group). **(D)** Relative levels of local IgE across various lymphoid tissues of B6 (red) or IgE KO (gray) mice, assessed by MFI of IgE bound to CD23 of FO B cells (CD19^+^ Fas^−^ CD38^+^ IgD^+^) as a proxy for local IgE concentration in the tissue. Numbers indicate relative IgE MFI, normalized to MFI of the corresponding inguinal LN and determined by flow cytometry (*n* = 1–12 mice/group, compiled from six independent experiments). **(E)** Percentage of true mLN IgE^+^ B cells (within live CD45^+^ c-Kit^−^ CD19^+^ B220^+^ population) of IgE reporter mice treated for 4 wk with Cont (gray) vs. DEX (red), as represented in the experimental setup (*n* = 8 samples/group with each point representing mean of two to three pooled mice, compiled from four independent experiments). **(F)** Representative flow cytometric gating for IgE^+^ B cells in mLNs of Cont (left) vs. DEX-treated (right) IgE reporter mice. True IgE-expressing cells were identified as double positive for fluorescent Venus (x axis) and anti-IgE stain (y axis) within live CD45^+^ c-Kit^−^ B220^+^ CD19^+^ population, as described in E (pooled from two to three mice, representative of four independent experiments). **(G)** Quantification by RNA-seq of mature *Ighe* transcripts and upstream εGLTs of sorted mLN B cells from mice treated for 4 wk with Cont (gray) or DEX (yellow). Numbers indicate CPM for *Ighe* (top) or RPKM from upstream regions of *Ighe* gene for εGLTs (bottom; *n* = 3 mice/group). **(H)** Hierarchical clustering by and relative expression of genes associated with plasmablast-like or germinal center and memory B cell–like signatures for sorted mLN B cells from Cont vs. DEX-treated mice. Color gradations indicate z-scores by CPM (*n* = 3 mice/group as biological replicates). **(I)** Percentage of IgE^+^ IgG1^−^ B cells (within live B220^+^) from splenic vs. mLN WT CD19^+^ B cells cultured with Cont (gray) or DEX (red), as described in Fig. 1 C (*n* = 3 mice/group). Error bars in A–D and G represent SEM. P values <0.05 were considered significant (*, P < 0.05; **, P < 0.01; ***, P < 0.001; ns, not significant). Welch’s *t* test was used for A–C and E, and ratio paired Student’s *t* test was used for D.

**Figure S3.**
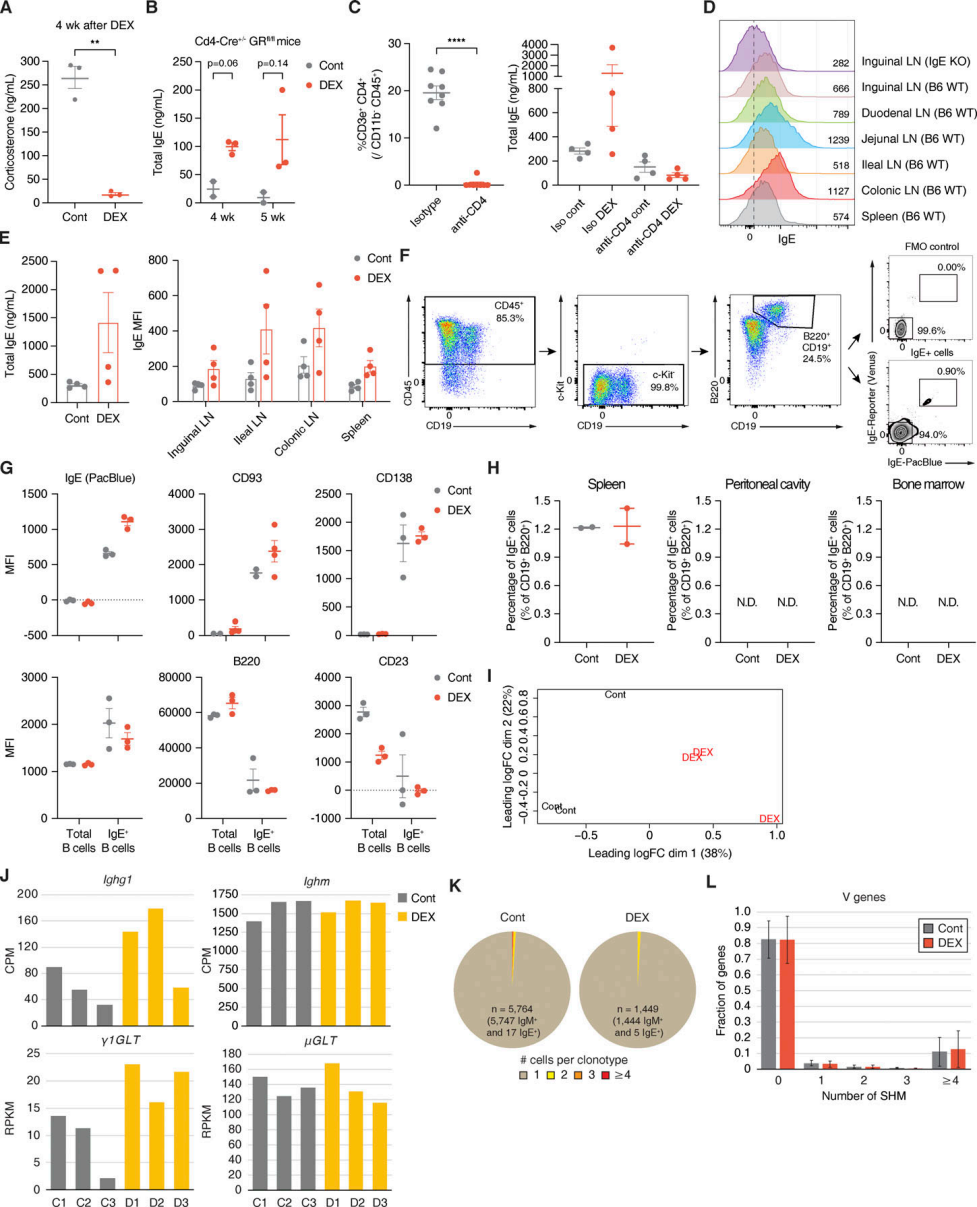
**Glucocorticoid-inducible natural IgE may be locally produced in mLNs. (A)** Level of plasma Cort at ZT4 after 4 wk of Cont (gray) vs. DEX (red) treatment (*n* = 3 mice/group). **(B)** Total plasma IgE concentration of *Cd4-Cre*^+/−^ GR KO mice after 4 or 5 wk of Cont (gray) vs. DEX (red) treatment. **(C)** Percentage of CD3e^+^ CD4^+^ T cells (within live CD45^+^ CD11b^−^) in mice injected i.p. with isotype control (gray) vs. anti-CD4 (red) antibody (left; *n* = 8 mice/group), and total plasma IgE concentration of isotype control vs. CD4-depleted mice after 2 wk of Cont (gray) or DEX (red) treatment (right; *n* = 4 mice/group). **(D)** Local IgE levels across various lymphoid tissues from representative IgE KO (row 1) vs. WT B6 (rows 2–7). Numbers on plot and in the legend indicate absolute IgE MFI of FO B cells (CD19^+^ Fas^−^ CD38^+^ IgD^+^), as determined by flow cytometry and described in Fig. 3 D. Dashed vertical line marks peak IgE KO value (representative of six independent experiments). **(E)** Total plasma IgE concentration (left) and corresponding local IgE levels by absolute FO B cell MFI across various lymphoid tissues (right) for WT mice treated for 4 wk with Cont (gray) vs. DEX (red; *n* = 4 mice/group). **(F)** Representative flow cytometric gating for a live CD45^+^ c-Kit^−^ B220^+^ CD19^+^ population, from which true IgE^+^ B cells were quantified from various lymphoid tissues of Cont vs. DEX-treated IgE reporter mice as described in Fig. 3, E and F. Stained samples were validated against their FMO controls for anti-IgE staining (pooled from two to three mice, representative of four independent experiments). **(G)** Surface protein expression for total or IgE^+^ mLN B cells from Cont (gray) or DEX-treated (red) IgE reporter mice. Numbers indicate MFI by flow cytometry within the appropriate CD19^+^ B220^+^ or CD19^+^ B220^+^ IgE^+^ populations, as described in F. **(H)** Percentage of true IgE^+^ B cells (within live CD45^+^ c-Kit^−^ CD19^+^ B220^+^) in spleen (left), peritoneal cavity (center), or bone marrow (right) of Cont (gray) vs. DEX-treated (red) IgE reporter mice (*n* = 2 samples/group with each point representing mean of two to three pooled mice). **(I)** Multidimensional scaling plot representing relative positions for sorted mLN B cells from Cont vs. DEX-treated mice. x and y axes represent leading log_2_(CPM fold change) for DEX/Cont in the first and second dimension, respectively (*n* = 3 mice/group as biological replicates). **(J)** Quantification by RNA-seq of mature *Ighg1* or *Ighm* transcripts (top) and upstream γ1GLTs or μGLTs (bottom) of sorted mLN B cells from Cont (gray) vs. DEX-treated (yellow) mice, as represented in Fig. 3, G–I. Numbers on plot indicate CPM for *Ighg1* or *Ighm* (top) or RPKM for γ1GLTs or μGLTs (bottom; *n* = 3 mice/group). **(K)** Clonotype abundance for mLN B cells of Cont (left) vs. DEX-treated (right) mice by single cell V(D)J sequencing. Sections represent clonotypes with one (brown), two (yellow), three (orange), or four (red) B cell clones (*n* = 5,764 B cells pooled from 21 Cont mice or *n* = 1,449 B cells pooled from 24 DEX-treated mice, compiled from three independent experiments). **(L)** Fraction of V region genes by number of SHM for mLN B cells of Cont (gray) vs. DEX-treated (red) mice, determined by single-cell V(D)J sequencing (pooled from 21 Cont or 24 DEX-treated mice, compiled from three independent experiments). Error bars in A–C, E, G, H, and L represent SEM. P-values < 0.05 were considered significant (*, P < 0.05; **, P < 0.01; ****, P < 0.0001; N.D., not detected). Welch’s *t* test was used for A–C and L.

Next, to assess whether glucocorticoid exposure affects in vivo antibody production, we treated WT C57BL/6J (B6; “wildtype”) mice with either DEX or vehicle control administered systemically through drinking water (Fig. 3 A and Fig. S3 A). Interestingly, WT mice exhibited elevated basal levels of plasma IgE at both 2 and 4 wk into DEX treatment (Fig. 3 A), while other antibody isotypes remained largely unaffected (Fig. 3 B). As basal IgE is generally low in naive WT mice and our otherwise unstimulated mice were housed in specific pathogen–free conditions, we surmise that the specificity of IgE induced by DEX reflects the broad specificity of “natural” IgE (McCoy et al., 2006). In contrast, cKO mice lacking GR only in B cells did not demonstrate elevated plasma IgE after 4 wk of DEX treatment, emphasizing the B cell–intrinsic nature of the DEX-dependent IgE response (Fig. 3 C). Notably, however, the B cell–specific deletion of GR did not impact the baseline level of IgE at steady state, suggesting that glucocorticoid-mediated IgE production is inducible rather than constitutively contributing to the basal IgE level (Fig. 3 C). Moreover, we confirmed that DEX was still able to increase the level of IgE in T cell–specific GR knockout mice (Fig. S3 B). However, we found that depletion of CD4^+^ cells that are thought to be the primary source of IL-4 actually abolished natural IgE induction in vivo (Fig. S3 C), which is consistent with an earlier study on natural IgE (McCoy et al., 2006). Given that the production of glucocorticoid-inducible IgE is not dependent on CD40L/CD40 signaling ex vivo (Fig. 2), it is possible that cognate T cell help may not be necessary for the synthesis of natural IgE despite the requirement for IL-4. However, this will need to be further investigated. These results show that glucocorticoid exposure increases the induction of IgE in vivo, and that such natural IgE induction by glucocorticoids is B cell–intrinsic and dependent on IL-4.

In addition, we sought to identify putative natural IgE-secreting B cells in vivo. Since IgE-producing cells are difficult to detect in vivo, we first took indirect approaches to understand where glucocorticoid-inducible IgE is produced. By analyzing the levels of surface IgE bound to the low-affinity receptor for IgE (CD23 or FcεRII) on follicular (FO) B cells as a proxy for local IgE concentration in a specific tissue, we observed that gut-draining LNs have higher levels of IgE than do skin-draining LNs or spleen (Fig. 3 D and Fig. S3 D; Materials and methods). This suggests that in B6 mice, IgE is produced primarily and locally in gut-draining mLNs at steady state, albeit at low levels. In line with this observation, DEX-treated mice maintained increased levels of bound IgE on FO B cells in ileal and colonic LNs, which was also correlated with the total IgE level in circulation (Fig. S3 E).

Furthermore, to directly identify natural IgE-secreting B cells in vivo, we utilized flow cytometric staining in tandem with IgE reporter mice that label IgE-expressing cells with fluorescent Venus protein (yellow fluorescent protein derivative; Yang et al., 2012). This approach enabled us to detect rare but true IgE^+^ B cells by circumventing potential false positive signals due to surface IgE bound to CD23 expressed on non-IgE^+^ B cells. Thus, we first treated IgE reporter mice for 2–4 wk with DEX or vehicle control, and then analyzed IgE expression in various lymphoid tissues by flow cytometry (Fig. 3 E and Fig. S3 F). DEX-treated mice exhibited a significant increase in IgE-expressing B cells from the mLNs (Fig. 3, E and F; and Fig. S3 G), which drain lymph from the small intestine and colon and are crucial for promoting immune tolerance to food antigens and commensal gut microbes (Houston et al., 2016). By contrast, we did not detect any significant IgE signal in the spleen, peritoneal cavity, or bone marrow of DEX-treated mice (Fig. S3 H). To confirm the expression of IgE in B cells from mLNs, we carried out total RNA-seq on mLN B cells sorted by FACS and found that εGLTs and mature transcripts of *Ighe* were highly induced by in vivo DEX treatment (Fig. 3 G and Fig. S3, I and J). Furthermore, these DEX-treated B cells in mLNs showed a plasmablast-like signature as compared with control B cells (Fig. 3 H and Table S3), which aligns with our observations from ex vivo work (Fig. 2 F). Interestingly, however, both splenic and mLN B cells had similar degrees of IgE switching when cultured with DEX ex vivo, implying that local environmental signals that are specific to the milieus of mLNs, rather than differential responsiveness to DEX, are crucial for triggering lgE switching in vivo (Fig. 3 I). Our findings thus present that B cells in mLNs preferentially produce natural IgE upon exposure to glucocorticoids.

Next, we examined the B cell receptor repertoire of mLN B cells by single-cell V(D)J sequencing. In line with previous reports on natural antibodies (Hayes et al., 2020; Kwon et al., 2022), we found that mLN B cells were polyclonal at steady state (Fig. S3 K). In addition to the diversity in clonotypes, SHM was rarely detected, suggesting that mLN B cells at steady state are unlikely to undergo a germinal center reaction (Fig. S3 L). Interestingly, these characteristics of mLN B cells remained unaffected by DEX, signifying that glucocorticoids alone do not lead to an alteration of the repertoire or SHM signature (Fig. S3, K and L). Given the absence of antigenic challenges in our model, our analyses thus suggest that glucocorticoids may increase the frequency of polyclonal IgE^+^ B cells in mLNs without affecting their repertoire.

### Glucocorticoids promote the production of natural IgE during gut dysbiosis

**Figure 4.**
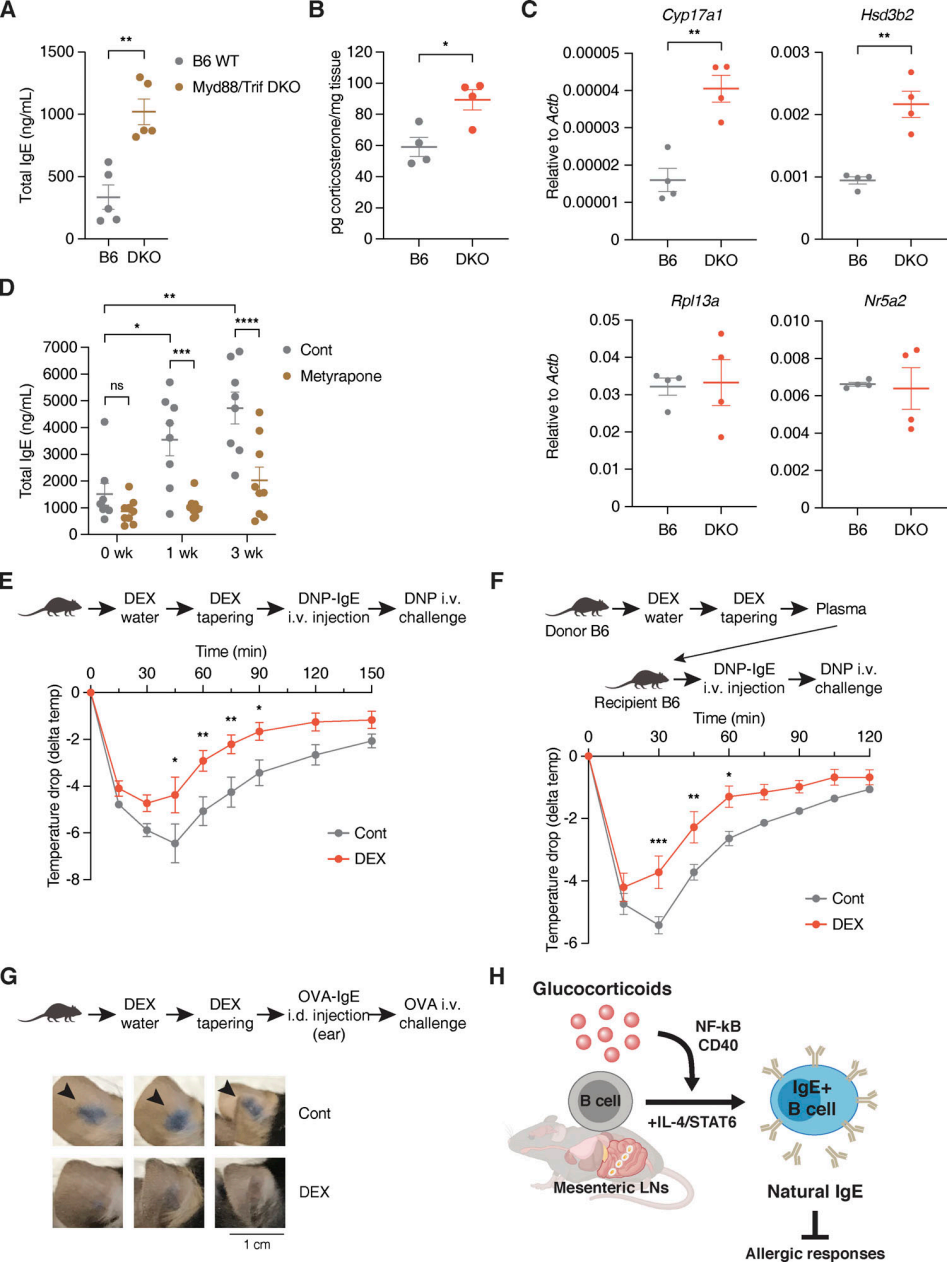
**Natural IgE is induced by glucocorticoids during dysbiosis and plays a protective role in allergic responses. (A)** Total plasma IgE concentration of 6 wk old B6 WT (gray) vs. age-matched DKO mice at steady state (brown; *n* = 5 mice/group, representative of three independent experiments). **(B)** Normalized level of Cort in the ileal epithelium (*n* = 4 male mice/group). **(C)** Relative RT-qPCR expression of genes involved in glucocorticoid synthesis in the ileal epithelium of B6 (gray) vs. age-matched DKO (red) mice. Expression was normalized by *Actb* (*n* = 4 male mice/group). **(D)** Total plasma IgE concentration of DKO mice at steady state (0 wk), or after 1 or 3 wk of Cont (gray) vs. metyrapone (brown) treatment. Mice were 6 wk of age at experiment start (*n* = 8–9 mice/group, compiled from two independent experiments). **(E)** Experimental setup and temperature drop time-course for assessing preemptive DEX treatment effects on passive systemic anaphylaxis using DNP-IgE and DNP-HSA. 3 wk old WT mice were treated for 4 wk with Cont (gray) or DEX (red) and tapered prior to anaphylaxis. x axis indicates time in minutes (min) after the challenge. y axis indicates temperature change in °C (delta temp) relative to baseline temperature at 0 min (total *n* = 11 mice/Cont group or *n* = 10 mice/DEX group with each point representing mean delta temp of two compiled and independent experiments). **(F)** Experimental setup and temperature drop time-course for passive systemic anaphylaxis after passive transfer of plasma from Cont (gray) or DEX-treated (red) WT mice (*n* = 5 mice/group). **(G)** Experimental setup and visual comparison of extravasation for assessing preemptive DEX treatment effects on passive cutaneous anaphylaxis using OVA-IgE and OVA. Arrows indicate Evans blue dye leakage in ears. A scale bar indicates 1 cm. 3-wk-old WT mice were treated for 4 wk with Cont or Dex and tapered prior to anaphylaxis (*n* = 3 mice/group). **(H)** Schematic model for the induction of “natural” IgE by glucocorticoids. Error bars in A–C represent SEM. P values <0.05 were considered significant (*, P < 0.05; **, P < 0.01; ***, P < 0.001; ****, P < 0.0001; ns [or unlabeled], not significant). Welch’s *t* test was used for A–D, and two-way ANOVA with post hoc Šídák’s multiple comparison test was used for E and F.

**Figure S4.**
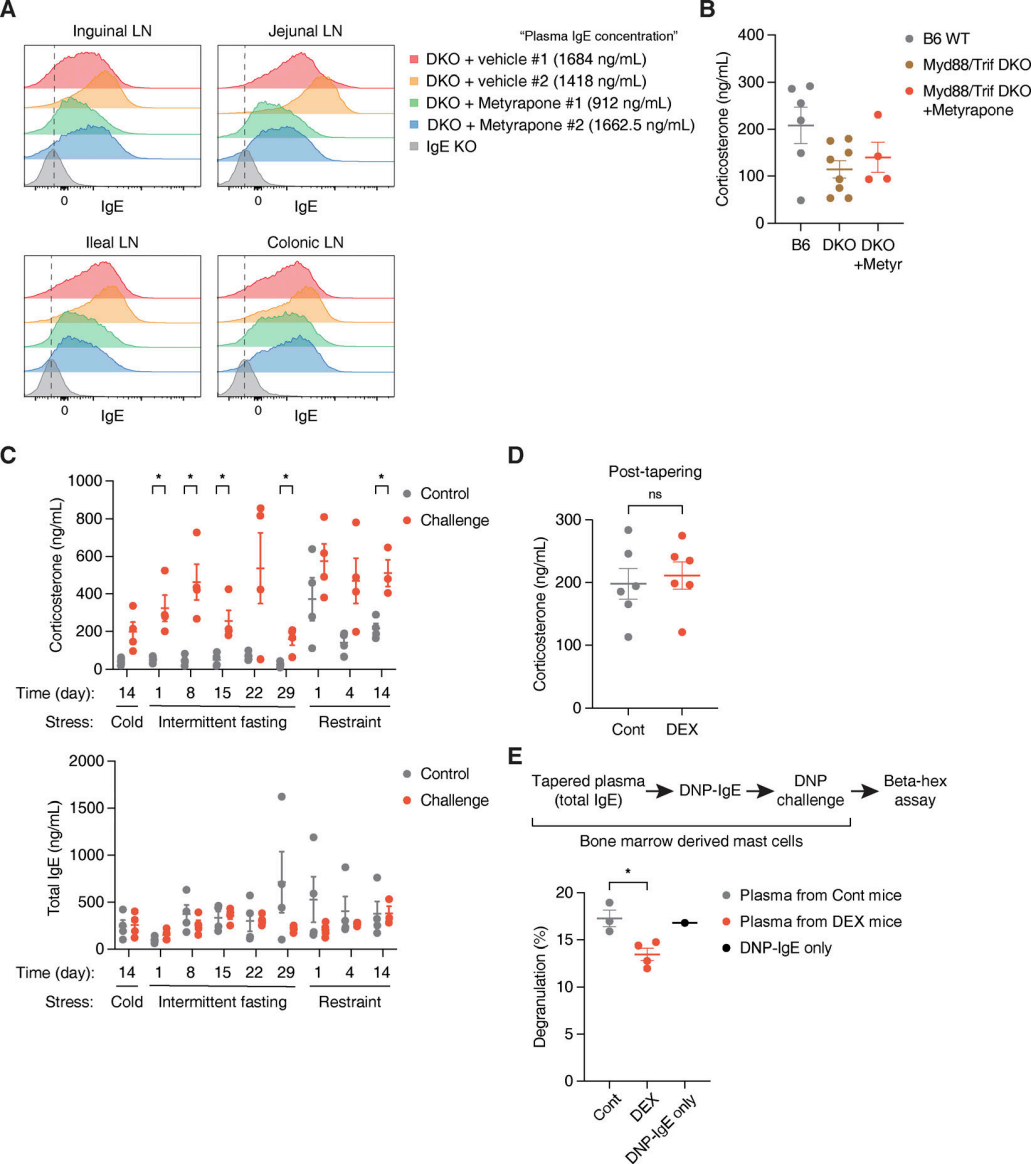
**Natural IgE is produced by local glucocorticoids in the gut, rather than by organismal stress conditions. (A)** Local IgE levels across various LNs from representative DKO mice after 3 wk of vehicle treatment (rows 1–2), DKO mice after 3 wk of metyrapone treatment (rows 3–4), and naive IgE KO mouse (row 5). x axis indicates absolute IgE MFI of FO B cells, as described in Fig. 3 D and Fig. S3 D. Corresponding systemic IgE concentrations are listed in the legend. Dashed vertical lines mark peak IgE KO values. **(B)** Plasma Cort concentration at ZT4 for naive B6 mice at steady state (gray), naive DKO mice at steady state or with Cont treatment (brown), or DKO mice treated for 1 wk with metyrapone (Metyr, red). B6 mice were 6 wk old upon bleeding, and DKO mice were 6–6.5 wk old at experiment start (*n* = 4–8 mice/group, compiled across two independent experiments). **(C)** Plasma Cort concentration (top) and total plasma IgE concentration (bottom) at ZT4 for WT mice throughout exposures to different physiological stress conditions. 6–8 wk old mice at experiment start were subjected to either no challenge (Control), cold challenge across 14 d, intermittent fasting across 29 d, or restraint stress across 14 d (*n* = 2–4 mice/group). **(D)** Level of plasma Cort at ZT4 after 4 wk of Cont (gray) vs. DEX (red) treatment followed by 2 wk of tapering (*n* = 6 mice/group). **(E)** Effects of bound natural IgE on bone marrow–derived mast cell degranulation ex vivo. Mast cells were preincubated without plasma (black) or with plasma from tapered Cont (gray) or DEX-treated (red) mice, and then induced to degranulate using DNP-IgE and DNP-HSA, as represented by the experimental setup. Numbers indicate percentage of degranulated mast cells, quantified by colorimetric assay for β-hex release (*n* = 4 mice/group, representative of two independent experiments). Error bars in B–E represent SEM. P values <0.05 were considered significant (*, P < 0.05). Unpaired Student’s *t* test was used for C and Welch’s *t* test was used for D and E.

We next asked in which physiological contexts local IgE induction can be licensed in mLNs by glucocorticoids. Given that immune cells in gut-draining LNs constantly interact with and rely on microbial signals to maintain gut homeostasis, disruption of gut homeostasis due to lack of microbial-induced signaling may trigger IgE elevation (Wesemann and Nagler, 2016). As previously reported, *Myd88*/*Trif* double-knockout (DKO) mice that lack the ability to respond to microbial signals showed markedly elevated plasma IgE (Fig. 4 A; Gavin et al., 2006). This suggests that gut perturbations or dysbiosis associated with the absence of microbial-derived signaling in DKO mice indeed contributes to IgE induction. We thus hypothesized that increased glucocorticoid production during dysbiosis may induce local natural IgE levels in mLNs to help protect from excess inflammation that could otherwise permit aberrant immune sensing, such as with food allergy (Renz et al., 2018). Indeed, we observed that DKO mice have higher levels of ileal Cort and expression of enzymes involved in glucocorticoid synthesis (Fig. 4, B and C). To further test the role of glucocorticoids in IgE induction during dysbiosis, we inhibited glucocorticoid synthesis in DKO mice (Fig. 4 D). At steady state, DKO mice exhibited elevated IgE levels that continued to rise through early adulthood (Fig. 4 D), an observation reminiscent of the spontaneous induction of circulating IgE that has been reported in germ-free mice (Cahenzli et al., 2013; Hong et al., 2019). Interestingly, suppression of glucocorticoid synthesis by metyrapone, which competitively inhibits the final 11β-hydroxylase (CYP11B1) enzyme of the biosynthetic pathway, significantly abolished the elevation of IgE in DKO mice (Fig. 4 D). Similarly, the local level of IgE in mLNs was reduced by metyrapone in DKO mice (Fig. S4 A). Metyrapone, however, did not decrease the systemic level of Cort in DKO (Fig. S4 B), suggesting that locally produced glucocorticoids in the gut milieu may be responsible for the induction of IgE in DKO mice. Related to this contrast between systemic and local glucocorticoids, we found that increased systemic Cort through physiological stress conditions was not sufficient to elevate basal IgE levels in WT mice (Fig. S4 C). We speculate that concomitant activation of the sympathetic nervous system in those stress conditions can lead to the inhibition of type 2 responses, including IgE synthesis (Florsheim et al., 2021). Therefore, circulating glucocorticoids produced by activation of the hypothalamic–pituitary–adrenal axis may not be responsible for the induction of natural IgE in vivo, which hints toward the importance of local production of glucocorticoids in the gut instead.

### Natural IgE may have a protective role in allergy

Lastly, we probed the physiological functions of natural IgE during allergy, when a substantial amount of high-affinity and antigen-specific IgE is also present. After 4 wk of treatment with DEX or vehicle control, followed by 2 wk of tapering to restore the immune system and adrenal functions, we induced a model of IgE-mediated passive systemic anaphylaxis with anti-dinitrophenyl (DNP) IgE (DNP-IgE) injection and DNP challenge (Fig. 4 E and Fig. S4 D). Upon DNP–human serum albumin (HSA) challenge, preemptively DEX-treated mice displayed reduced hypothermia, which resulted in faster recoveries to baseline temperature that are suggestive of less severe anaphylaxis as compared with “allergic” control mice (Fig. 4 E). Similarly, we observed that as compared with mice receiving control plasma, naive B6 mice passively receiving plasma from DEX-treated and tapered mice displayed reduced hypothermia and faster recoveries from DNP-IgE–mediated passive systemic anaphylaxis (Fig. 4 F). We also observed consistent results from a model of passive cutaneous anaphylaxis with anti-OVA IgE (OVA-IgE) injection and OVA challenge, whereby preemptively DEX-treated mice demonstrated reduced accumulation of Evans blue dye in the ear as a proxy for vasodilation and fluid extravasation during anaphylaxis (Fig. 4 G). In line with our in vivo findings, we also detected a reduction in DNP-IgE–induced degranulation of ex vivo bone marrow–derived mast cells that were preemptively incubated with IgE from DEX-treated and tapered mice (Fig. S4 E). Although we cannot fully exclude a contribution from IgG antibodies, natural IgE that is preemptively induced by glucocorticoids can play a physiological role in dampening or protecting from subsequent allergic symptoms, presumably by competing for FcεRI binding with high-affinity allergen-specific IgE (Erb, 2007; Oettgen, 2016).

## Discussion

We report that in the absence of experimentally administered antigens, glucocorticoids spontaneously induce “natural” IgE by acting directly on B cells to promote IgE switching both ex vivo and in vivo (Fig. 4 H). Given that the IgE-promoting effect of glucocorticoids is not reliant on CD40L/CD40 signaling (Fig. 2), cognate T cell help may not be required for the synthesis of natural IgE in vivo. This is consistent with an earlier study showing both antigen-independent and MHC class II–independent production of natural IgE (McCoy et al., 2006). Our findings, therefore, highlight the importance of soluble factors, rather than direct cellular interaction with antigen presentation, for the induction of natural IgE.

Moreover, we present that the natural IgE antibodies induced by glucocorticoids may represent a polyclonal repertoire with minimal SHM. Similar to other types of natural antibodies, glucocorticoid-inducible IgE may also recognize endogenous antigens with probable broad specificity and low affinity. This is in line with our results which show that glucocorticoids may promote direct IgE switching from IgM to IgE, which generates low-affinity IgE (Fig. 1 D and Fig. 2 G). It will be interesting to identify antigens that natural IgE recognizes. However, as antigen presentation is not necessary for the induction of natural IgE (McCoy et al., 2006) and affinity maturation with SHM is unlikely to occur, it is also plausible that natural IgE might arise and function independently of antigens, or at least as limited to certain classes of endogenous antigens.

Still, it remains elusive how such polyclonal IgE mitigates allergic responses. Prior studies have shown that sensitizing mice with both high- and low-affinity IgE to allergen protects mice from subsequent anaphylaxis (Xiong et al., 2012), and adding excess recombinant Fc fragment that binds FcεRI can inhibit antigen-specific IgE reactions (Geha et al., 1985). Furthermore, removing sialic acid glycans has been shown to perturb the anaphylactic ability of IgE (Shade et al., 2020). Given that our DEX-treated B cells demonstrate differences in levels of glycosylation enzymes like *St3gal1* (Fig. 2 C), natural IgE could harbor distinct glycan modifications that alter affinity for FcεRI or dampen downstream FcεRI signaling once IgE is bound (Jennewein and Alter, 2017; Shade et al., 2020). Perhaps excess amounts of low-affinity natural IgE, through potential higher affinity for FcεRI, can compete with and outbind allergen-specific IgE for FcεRI sites on mast cells or basophils. Alternatively, even with a similar affinity for FcεRI, natural IgE could also differentially affect the downstream FcεRI signaling pathway, thereby altering the degranulation or activation state of mast cells and basophils.

Our work also provides a potential link between stress and type 2 immunity. Although stress hormones are typically considered to suppress the immune system, including type 2 immune responses, our data suggest that glucocorticoids can indeed positively regulate B cells to secrete natural IgE. Then, the question remains whether any type of stress can promote the induction of IgE. As we demonstrated that glucocorticoids produced by organismal stress conditions are incapable of inducing IgE (Fig. S4 C), systemic glucocorticoids that derive from adrenal glands may not be major contributors to natural IgE production in vivo. Instead, extra-adrenal glucocorticoids that are produced in the ileum by local stress conditions may be responsible for the induction of natural IgE (Ahmed et al., 2019). We speculate that these local stress conditions may be associated specifically with triggers of type 2 immune responses, such as the disruption of epithelial barriers and perturbation of gut homeostasis.

In the future, it would be important to identify physiological and pathophysiological conditions that increase the local level of glucocorticoids in the gut, perhaps in tandem with altered gut immune responses. It has previously been shown that depletion of microbiota is associated with the induction of ileal glucocorticoids (Mukherji et al., 2013), suggesting that microbial signals restrain glucocorticoid levels during homeostasis. In addition, inflammation and epithelial cell damage can induce extra-adrenal synthesis of glucocorticoids primarily in the ileum via the orphan nuclear receptor LRH-1 (liver receptor homolog-1 or NR5A2; Ahmed et al., 2019; Mueller et al., 2006). Likewise, toxins, noxious chemicals, and xenobiotics, which can trigger type 2 immune responses, may cause sufficient inflammatory tissue damage to induce glucocorticoid production in the intestinal epithelium that further impacts mucosal immunity. Thus, “local stress” caused by the perturbation of gut homeostasis may be attributed to the induction of local glucocorticoids, and thereby of natural IgE. Accordingly, natural IgE triggered by such local stresses may play a pivotal role in maintaining gut homeostasis and preventing the onset of aberrant immune sensing, such as with food allergy (Renz et al., 2018).

In line with the concept of local glucocorticoid production, we also demonstrate that rare B cells in mLNs are responsible for the production and secretion of natural IgE. Studies have shown that there is likely more immune activity in mLNs than non-gut-draining LNs at steady state (Casola et al., 2004; Hoshi et al., 2001; Kunieda et al., 2002). It remains unclear, however, which tissue-specific properties set mLNs apart as a preferential and particularly conducive milieu for IgE switching. For example, IL-4 as well as glucocorticoids may be more readily available in the mLNs as compared to other lymphoid tissues. Given that depletion of CD4-expressing cells that are major IL-4 producers eliminates natural IgE while depletion of GR in those cells does not (Fig. S3, B and C), IL-4 production is not dependent on glucocorticoids and may remain constant instead. Potential constitutive production of IL-4 in the gut, which may arise from homeostatic interactions with microbiota or diet, could prime mLNs as a privileged tissue for the production of natural IgE. Similarly, it is possible that differential dynamics of IL-4–producing cells around mLNs also permit enhanced IL-4 availability for mLN B cells that are readily poised, as with glucocorticoid exposure, to undergo switching and produce natural IgE. These would be consistent with our observations that purified B cells from mLNs and spleen show similar responsiveness to DEX ex vivo in the presence of IL-4 (Fig. 3 I).

Furthermore, although we have shown that DEX increases mLN εGLTs, indicative of increased IgE switching, how natural IgE-producing B cells home after class switching and differentiation has yet to be fully elucidated. On one hand, as supported by our findings that IgE is produced primarily and locally in mLNs at steady state in B6 mice and can be further induced by DEX, mature naive B cells trafficking through mLNs and undergoing more frequent IgE switching in response to DEX may remain resident to mLNs, thereafter, leading to greater accumulation of natural IgE-secreting plasmablasts in mLNs of DEX-treated mice. By contrast, given that GR signaling has been shown to regulate CXCR4-mediated B cell homing between blood and bone marrow (Cain et al., 2020), mature naive B cells trafficking through mLNs and undergoing more frequent IgE switching in response to DEX may not accumulate in mLNs as resident IgE^+^ B cells, but instead, continue trafficking between lymphoid tissues as plasmablasts with a homing preference for mLNs. Therefore, it will be interesting to investigate the migration dynamics and differentiation kinetics of IgE^+^ B cells in mLNs upon glucocorticoid exposure.

While our findings seem to oppose long-standing perspectives on the classically immunosuppressive effects of glucocorticoids, recent studies have similarly found that glucocorticoids have dose-dependent effects on B and T cells that can be immunoenhancing at particular ranges (Cain et al., 2020; Hong et al., 2020; Shimba et al., 2018). Further, consistent with our findings, induction of NF-κB signaling by glucocorticoids has also been identified by others. For example, low-dose Cort increases NF-κB translocation and downstream proinflammatory gene transcription in microglia-like immune cells (Liu et al., 2018). Indeed, we observed that in addition to promoting IgE switching by activating NF-κB to reinforce CD40L/CD40 signaling, glucocorticoids predispose ex vivo B cells to become plasmablast-like cells with distinct transcriptomes enriched for genes like *Prdm1* and *Xbp1*, as revealed by RNA-seq. Thus, our data further bolster the immunoenhancing role of glucocorticoids in B cells by activating NF-κB, promoting a plasmablast-like fate, and inducing downstream IgE production.

However, we also observed that despite IgE induction, the immune system was otherwise systemically suppressed by DEX treatment as we used a supraphysiological dose in our experiments (Materials and methods). In addition to our present findings on glucocorticoid induction of IgE synthesis in B cells, prior literature has reported higher IgE levels in patients receiving multiple high-dose corticosteroid treatments for asthma, despite reduced IFN-γ–producing T cells and natural killer cells as proxies for immunosuppression (Zieg et al., 1994). Thus, given that the valence of glucocorticoid regulation of immune cells is cell type specific, dose dependent, and sensitive to cues from the surrounding tissue milieu, it is probable that glucocorticoids can exhibit immunoenhancing effects on particular immune cell populations, despite a net state of immunosuppression in the whole organism. It would be particularly interesting to investigate in which physiological contexts glucocorticoids can positively regulate immune responses, and whether GR-mediated changes are accompanied by epigenetic modifications depending on the specific cell type.

In all, our study collectively suggests that glucocorticoids act as promoting factors for natural IgE production and provides important mechanistic insights into the induction and protective physiological functions of glucocorticoid-induced IgE. Moreover, our results suggest that chronic glucocorticoid treatment may potentially increase IgE levels in humans, which has indeed been previously reported for the aforementioned patients who received long-term prednisone treatment (Zieg et al., 1994). Additional studies are required to determine whether the same pathway identified in our work is similarly responsible for IgE induction in humans. Furthermore, understanding the immunoenhancing effects of glucocorticoids on B cells opens up multiple new avenues for the design of novel therapeutics for allergy and other IgE-mediated hypersensitivity conditions. For example, prophylactic local glucocorticoid treatments may alleviate allergic responses in the gut against food allergens. In addition, passive administration of recombinant natural IgE antibodies could be used as therapeutics for allergic patients. Thus, our work not only establishes a mechanistic link between glucocorticoids and elevated natural IgE but also sets the stage for novel approaches to treat allergies afflicting much of our world today.

## Materials and methods

### Mice

C57BL/6J WT (B6, 000664), *Stat6* knockout (005977), *Cd19-Cre* (006785), *Cd4-Cre* (022071), and *Nr3c1*^fl/fl^ (GR^fl/fl^, 021021) mice were purchased from Jackson Laboratory and bred in-house. *Mb1-Cre* mice were a kind gift from Dr. Joao Pereira at Yale (New Haven, CT). *Mb1-Cre* or *Cd19-Cre* mice were crossed with *Nr3c1*^fl/fl^ mice to yield *Mb1-Cre*^+/−^ or *Cd19-Cre*^+/−^ *Nr3c1*^+/+^, *Nr3c1*^fl/+^, and *Nr3c1*^fl/fl^ mice (B cell–specific *Nr3c1 WT*, Het, and KO [cKO]). *Stat6*^−/−^ mice were crossed with B6 mice to yield *Stat6*^+/−^ mice, which were crossed to yield *Stat6*^+/+^, *Stat6*^+/−^, and *Stat6*^−/−^ mice. *Cd4*-*Cre* mice were crossed with *Nr3c1*^fl/fl^ mice to yield *Cd4-Cre*^+/−^ *Nr3c1*^fl/fl^ mice (T cell–specific *Nr3c1* KO). Breeders of *Myd88/Trif* KO mice (DKO) were maintained on a sulfamethoxazole/trimethoprim antibiotic diet every other 2 wk (Rakoff-Nahoum et al., 2015). IgE KO B6 mice were generated by backcrossing 10 times from IgE KO Balb/cJ mice that were generously donated by Dr. Hans Oettgen at the Boston Children’s Hospital (Boston, MA). B6 Venus IgE reporter mice (Verigem) were a kind gift from Dr. Christopher Allen at the University of California, San Francisco (San Francisco, CA). All mice were female, 3–6.5 wk old at experiment start, littermates unless otherwise stated, and maintained in a specific pathogen–free facility. Mice were bred and maintained at the Yale Animal Resources Center at Yale University in specific pathogen–free conditions. All animal protocols were reviewed, approved, and conducted under the institutional regulation of Yale University’s Institutional Animal Care and Use Committee.

### Splenic B cell and lymphoid tissue harvest

For CD19^+^ splenic B cell isolation, spleens of WT B6, *Stat6*, or cKO mice were harvested, then mashed through a 70-µm strainer (087712; Thermo Fisher Scientific) into a 6-well flat-bottom plate containing “complete media” (RPMI-1640 media with 2 μM L-glutamine [R8758; Sigma-Aldrich; 21875034; Thermo Fisher Scientific], further supplemented with 15% FBS [10438026; Gibco or 100-106; BenchMark or S11550; R&D Systems], 100 U/ml penicillin and 100 µg/ml streptomycin [P4333; Sigma-Aldrich], and 50 µM 2-ME [M6250; Sigma-Aldrich]). Erythrocytes were lysed by 1× ammonium-chloride-potassium lysis buffer (BP10-548E; Lonza). Pellets were washed with 1× magnetic-activated cell sorting (MACS) buffer (1× PBS [14190144; Gibco] with 0.5% BSA [BP1600-100; Thermo Fisher Scientific] and 2 mM EDTA [AM9260G; Invitrogen]). CD19^+^ B cells were positively selected by MACS using mouse CD19 MicroBeads (130-121-301; Miltenyi Biotec) and LS columns (130-042-401; Miltenyi Biotec) on a MACS MultiStand (130-042-303; Miltenyi Biotec), all following the manufacturer’s protocol. Duodenal, jejunal, ileal, colonic, inguinal, and axillary LNs were harvested, mashed through a 70-µm strainer into ice-cold 1× PBS with 2.5% FBS, and incubated with 1× ammonium-chloride-potassium lysis buffer, prior to flow cytometry, FACS, or RNA-seq preparation. Specifically, mLNs were pooled from harvested duodenal, jejunal, ileal, and colonic LNs. Peritoneal cells and bone marrow, isolated by peritoneal lavage and flushing of bones, respectively, were washed prior to flow cytometry preparation.

### Ex vivo B cell culture

We adopted a protocol from Vaidyanathan et al. (2017) for IgE and IgG1 B cell class switch recombination. Purified splenic CD19^+^ B cells of WT, cKO, *Stat6* knockout, or Verigem IgE reporter mice, as well as purified WT mLN CD19^+^ B cells were seeded in 6-well flat-bottom plates at 8.75 × 10^5^ cells/ml in complete media containing 1 µg/ml anti-mouse CD40 monoclonal antibody (aCD40; 553721; BD Biosciences) and 10 ng/ml IL-4 (404-ML; R&D Systems). For a 6 h incubation from 48 to 72 h, cells were resuspended in 12-well flat-bottom plates at 10^6^ cells/ml either in complete media with 0.5 µg/ml aCD40 and 5 ng/ml IL-4, or in SF media lacking FBS but otherwise containing all remaining complete media components with 0.5 µg/ml aCD40 and 5 ng/ml IL-4. During this 6-h window, cells were additionally incubated with either 0.1% DMSO (AB03091; AmericanBio) or PBS as a control; 100 ng/ml Cort (diluted in DMSO; C2505; Sigma-Aldrich); 100 ng/ml DEX (diluted in DMSO; D1756; Sigma-Aldrich); 1 mg/ml RU-486 (diluted in DMSO; M8046; Sigma-Aldrich); 2% 50X B-27 SF supplement or its variants (17504044, 10889038, 12587010, or A1895601; Thermo Fisher Scientific); 15% “boiled” FBS incubated for 15 min at 90°C; 0.2% chemically defined lipid mixture (CDL or CDLipid; L0288; Sigma-Aldrich); 3.15 ng/ml progesterone (Prog; P8783; Sigma-Aldrich); 1 ng/ml 3,3′,5-triiodo-*L*-thyronine (T3; T2877; Sigma-Aldrich); or 20 µg/ml TPCA-1 (ab145522; Abcam). An identical 6-h incubation was repeated from 72 to 78 h while maintaining similar cell density. Outside of the two 6-h incubations up through the 96-h time point, washed cells were resuspended in complete media with 0.5 µg/ml aCD40 and 5 ng/ml IL-4. For IL-4 or CD40L (aCD40) depletion experiments, cells were cultured from 0 to 48 h in complete media containing 10 ng/ml IL-4 and 1 µg/ml aCD40, then cultured from 48 to 96 h with or without appropriate DEX incubations in complete media containing 0.5 µg/ml aCD40, 5 ng/ml IL-4, or both 0.5 µg/ml aCD40 and 5 ng/ml IL-4. For cell proliferation labeling, B cells were stained prior to culture with the CellTrace Violet Cell Proliferation Kit (C34571; Thermo Fisher Scientific) as per manufacturer protocol and assessed for cell divisions after 96 h by flow cytometry. For mLN B cell cultures, mLNs were subjected to anti-CD19 MACS, as described above, and then cultured ex vivo in identical conditions as for splenic B cells. All cultures were maintained in a 37°C incubator at 5% CO_2_.

### Flow cytometry and analysis

All antibodies for flow cytometry are shown in Table S4 and were titrated based on manufacturer protocols. For the preparation of ex vivo cultures at 0-, 48-, 72-, or 96-h time points, we adapted a protocol from Wesemann et al. (2011) to detect true IgE^+^ B cells by initially cleaving off surface FcεRII-IgE complexes before staining for intracellular IgE proteins, thus avoiding false signal from IgE^−^ B cells with IgE bound to surface CD23 (FcεRII). In brief, harvested B cells were initially incubated for 1 min with 0.1% trypsin-EDTA (25200056; Gibco), neutralized with ice-cold FBS and 1× PBS, then assessed for viability using Zombie Red or Zombie Yellow Fixable Viability Kit (423110 or 423103; BioLegend). Cells were then fixed and permeabilized with Foxp3/Transcription Factor Staining Buffer Set (00-5523-00; eBioscience), followed by intracellular staining with fluorochrome-conjugated antibodies and anti-CD16/32 Fc block (14-0161-86; eBioscience) according to the Staining Buffer protocol. In particular, permeabilized PBMCs from cKO mice were verified for GR deletion using monoclonal unconjugated rabbit anti-mouse GR antibody (12041; Cell Signaling), followed by polyclonal goat anti-rabbit IgG (H+L) antibody conjugated to Alexa Fluor 488 (A-11034; Invitrogen). For the preparation of harvested in vivo tissues, cells were optionally assessed for viability using Zombie Red or Zombie Yellow, followed by surface staining with fluorochrome-conjugated antibodies diluted in 1× PBS with 2.5% FBS and Fc block. For all samples following viability and/or antibody staining, cells were washed, resuspended in ice-cold 1× PBS with 2.5% FBS, and filtered through 80 µm nylon mesh. FACS data were collected on a LSR II Flow Cytometer (BD Biosciences) and analyzed by FlowJo 9 (Tree Star). B cell populations were identified by gating on singlet, live, and B220^+^ (also known as CD45R). Specifically, FO B cells were identified by sequentially gating on singlet, live, CD19^+^ FcεRI^−^, Fas^−^, CD38^+^ IgD^+^ cells, and analyzed for median fluorescence intensity (MFI) for IgE. For true IgE^+^ B cell identification with IgE reporter mice, singlet, live, CD45^+^, c-kit^−^, B220^+^, and CD19^+^ cells were analyzed for surface expression of IgE and reporter Venus signal. Stained samples were validated against fluorescence minus one (FMO) controls. This approach thus excluded mast cells or basophils with IgE bound to FcεRI and non-IgE-producing B cells with IgE bound to CD23.

### RT-qPCR

Total RNA was extracted by RNeasy Micro Kit (74004; Qiagen) with on-column DNase treatment (79256; Qiagen) or by Direct-zol RNA Miniprep (R2052; Zymo). Reverse transcription was done with random hexamer primers and SMART MMLV Reverse Transcriptase (639524; Clontech). RNA levels were measured with PerfeCTa SYBR Green SuperMix, Low ROX (95056-02K; Quantabio), and CFX96 Touch Real-Time PCR Detection System (Bio-Rad). The list of RT-qPCR primers is shown in Table S5.

### RNA-seq and analysis

IgE^+^ B cells (Venus IgE reporter positive) from ex vivo B cell cultures treated with DEX or DMSO, as described above, or B cells (gated on singlet live CD45^+^ c-Kit^−^ B220^+^) from mLNs of DEX or vehicle-treated mice were sorted by FACS through Yale Flow Cytometry Facility. Total RNA was isolated by RNeasy Micro Kit (74004; Qiagen). RNA-seq libraries were prepped by SMARTer Stranded Total RNA-Seq Kit v2-Pico Input Mammalian (634413; Takara). The libraries were sequenced on NextSeq 500 (Illumina) by paired-end run (38 × 38 or 42 × 42 bp). Three nucleotides from 5′ end of read 2 were trimmed by FASTX-Toolkit (http://hannonlab.cshl.edu/fastx_toolkit/). Reads were mapped to mouse cDNA/ncRNA transcriptomes (GRCm38 ensembl v89) and quantified in transcripts per million with kallisto (v0.46.2, k-mer index 25, bootstrap 60; Bray et al., 2016). Differential gene expression analysis was conducted in RStudio (v4.1.0) with biomaRt, tximport, edgeR, Glimma, and limma packages with generalized linear model likelihood ratio test and robust gene-wise dispersion parameter estimates. Pathway analysis was conducted by ingenuity pathway analysis (Qiagen). We also aligned the reads to the mm10 genome by STAR (v2.5.3) and observed similar results. To quantify the epsilon, gamma1, and mu germline transcripts (εGLTs, γ1GLTs, and μGLTs), we calculated reads per kilobase of transcript per million mapped reads (RPKM) from the upstream regions of immunoglobulin genes (*Ighe*: chr12:113,273,506–113,277,355; *Ighg1*: chr12:113,338,827–113,339,438 and chr12:113,334,226–113,337,296; and *Ighm*: chr12:113,422,854–113,424,477). Sequencing data have been deposited in the National Center for Biotechnology Information Gene Expression Omnibus database (accession no. GSE203542).

### Western blotting

Cells were lysed in 1% radioimmunoprecipitation assay buffer (9806S; Cell Signaling Tech), and 15 µg of lysate was loaded on 4–15% Mini-PROTEAN TGX Precast Protein Gels (4561084; Bio-Rad). After transferring to a methanol-activated polyvinylidene difluoride membrane (1620175; Bio-Rad) using Trans-Blot Turbo Transfer System (Bio-Rad), the membrane was blocked in 0.1% Tween 20 (P9416; Sigma-Aldrich) in 1× PBS containing 5% BSA, probed with primary antibodies listed below, and washed three times. The membrane was then incubated with anti-mouse StarBright Blue 700 (12004158; Bio-Rad) or anti-rabbit HRP-conjugated secondary antibodies (18-8816-33; Rockland) before washing an additional three times. Detection was performed with West Pierce ECL Substrate (32106; Thermo Fisher Scientific), and chemiluminescent signals were detected by ChemiDoc Imaging System (Bio-Rad). Anti-IκBa (1:1,000, sc-371; SantaCruz), anti-IRF4 (1:1,000, 62834T; Cell Signaling Tech), or anti-β-Actin (1:2,000, 3700S; Cell Signaling Tech) were used as primary antibodies.

### In vivo drug treatment

For DEX treatment, WT, cKO, or IgE reporter mice were treated for 2 or 4 wk with 2-hydroxypropyl-β-cyclodextrin (2-HCD; H107; Sigma-Aldrich) as a vehicle or with 1.3 µg/ml DEX diluted in 0.02% DMSO and balanced with 0.0018% 2-HCD vehicle in autoclaved drinking water (200 μl DMSO or DEX first mixed with 1,800 μl 1% 2-HCD, all dissolved in 1 liter water). We treated mice by oral water consumption rather than injection to mitigate stress and estimated that mice received 0.36 mg/kg DEX per day, delivered systemically (Hong et al., 2020; Yang et al., 2009). For metyrapone treatment, *Myd88*/*Trif* DKO mice were treated for 3 wk with 2-HCD vehicle or with 0.8 mg/ml metyrapone (14994; Cayman) diluted in 0.16% DMSO and balanced with 0.0034% 2-HCD. Treatments were provided in water bottles and replenished every 7–10 d, and automatic water valves were removed for treated cages. For the depletion of CD4^+^ cells, B6 mice were injected i.p. with 1 mg of anti-CD4 antibody (BE0003-1; Bio X Cell) or isotype control (Bio X Cell, BE0090; Bio X Cell).

### Plasma isolation

Whole blood was harvested from mice by retroorbital bleeding. To isolate plasma, harvested blood samples were spun based on the manufacturer protocol in BD Microtainer PST tubes with lithium heparin (365985; BD Biosciences). Isolated plasma samples were either analyzed immediately or stored at −80°C until assessment.

### ELISA

To assay for total plasma IgE, 96-well Nunc MaxiSorp plates (44-2404-21; ThermoFisher) were coated overnight at 4°C with 2 µg/ml rat anti-mouse IgE (553413; BD Biosciences) diluted in 0.1 M pH 9.6 carbonate-bicarbonate buffer (C3041; Sigma-Aldrich). Plates were washed in 1× PBS with 0.05% Tween 20, blocked in 1× PBS with 1% BSA at room temperature for 1 h, and then incubated for 2 h at room temperature with plasma or serially diluted mouse IgE κ isotype control (557080; BD Pharmingen), all diluted in 1× PBS with 1% BSA. For detection, the washed plates were incubated with 0.5 µg/ml biotinylated rat anti-mouse IgE (553419; BD Biosciences) for 1 h at room temperature, washed, and incubated for 30 min at room temperature with streptavidin–HRP (1:1,000, 554066; Thermo Fisher Scientific), all diluted in 1× PBS with 1% BSA. The washed wells were developed with TMB substrate solution (555214; BD Biosciences), quenched with 3 M H_2_SO_4_ (258105; Sigma-Aldrich), and detected at 450 nm by spectrophotometer for optical density and back-calculated concentration (SpectraMax M5). Other antibody isotypes were assayed with the LEGENDplex Mouse Immunoglobulin Isotyping Panel by flow cytometry (740493; BioLegend). Cort levels from plasma collected at Zeitgeber time 4 (ZT4) or from cell lysates of ileal epithelium were assayed by the DetectX Corticosterone Enzyme Immunoassay Kit (K014-H1; Arbor).

### In vivo passive systemic anaphylaxis

After 4 wk of vehicle or DEX treatment as described above, B6 mice were tapered back to drinking water over 2 wk. For the plasma transfer experiment, plasma from such control or DEX-treated and tapered mice were pooled and injected i.p. into B6 recipient mice. Mice were then injected i.v. with 2 µg monoclonal anti-DNP IgE (DNP–IgE, clone SPE-7; D8406; Sigma-Aldrich) diluted in 100 μl 1× PBS. After 24 h, mice were challenged i.v. with 20 µg DNP-HSA (D-5059; Biosearch Technologies) diluted in 100 μl 1× PBS. Following DNP-HSA challenge, mice were probed for rectal temperature across time (TH-5 and RET-3; Physitemp).

### In vivo passive cutaneous anaphylaxis

After 4 wk of vehicle or DEX treatment as described above, B6 mice were tapered back to drinking water over 2 wk. Mice were then injected intradermally with 20 ng monoclonal anti-OVA IgE (clone E-C1; 7091; Chondrex). After 24 h, mice were challenged i.v. with 20 µg grade V OVA (A5503; Sigma-Aldrich) mixed with 1% Evans Blue dye (E2129; Sigma-Aldrich). After 30 min, mice were euthanized and assessed for Evans blue dye leakage in the ear, indicative of vascular permeability and histamine-driven extravasation during anaphylaxis.

### Quantification and statistical analysis

Error bars represent SEM, and each dot represents a sample from an individual mouse unless otherwise stated. As noted in figure legends and deemed appropriate by experimental design, statistical analyses were determined by two-tailed parametric unpaired Student’s *t* test or Welch’s *t* test, ratio paired Student’s *t* test, one-way ANOVA with post hoc Tukey’s multiple comparisons test, or two-way ANOVA with post hoc Šídák’s multiple comparisons test (full model mixed-effects analysis). P < 0.05 was considered statistically significant. Tests were conducted with GraphPad Prism 9.0 (GraphPad Software, Inc).

### ATAC sequencing (ATAC-seq) and analysis

ATAC-seq libraries were constructed with 5 × 10^4^ cells from each condition following Omni-ATAC protocol (FC-121-1031; Illumina; Corces et al., 2017). Libraries were sequenced on Illumina HiSeq 2,000 (paired-end run, 100 × 100 bp) by Yale Center for Genome Analysis (YCGA). Sequenced reads were trimmed with adaptor sequences (cutadapt v1.9.1; Martin, 2011) and mapped to the mouse genome (GRCm38, ensembl release 93) by Bowtie2 (v2.3.4.1; Langmead and Salzberg, 2012). Mitochondrial and duplicated reads were removed by SAMtools (v1.9; Li et al., 2009) and Picard (v2.9.0, https://broadinstitute.github.io/picard/), respectively. Peaks were found by MACS2 (v.2.1.1; Zhang et al., 2008) and visualized by deepTools (v3.1.1; Ramirez et al., 2014).

### Single cell V(D)J sequencing and analysis

mLN B cells (CD45^+^ FcεRI^−^ B220^+^ CD23^lo^ or CD45^+^ FcεRI^−^ c-kit^−^ B220^+^ CD19^+^) were sorted from B6 control or DEX-treated mice and subjected to 10× Chromium Single Cell 5′ Reagent Kits v2. Gene expression libraries and V(D)J libraries were constructed by YCGA. The libraries were sequenced on NovaSeq 6000 (paired-end run, 26 × 90 bp). Raw sequencing data were analyzed by Cell Ranger (v6.1.2) using mm10-2020-A and GRCm38-alts-ensembl-5.0.0. Clonotypes were analyzed by Loupe VDJ Browser 4.0, and the frequency of SHMs was analyzed by AssignGenes and MakeDB python packages of the Change-O (v1.0.0).

### Organismal stress conditions

For a cold challenge, mice were housed at 4°C for 14 d. For the intermittent fasting challenge, mice were fasted overnight every 1–2 d over a period of 29 d. For restraint stress, mice were restrained for 4–6 h in 50 ml conical tubes every 1–2 d over a period of 14 d.

### In vitro mast cell degranulation (β-hexosaminidase [β-hex]) assay

To assess mast cell degranulation, we adapted a published protocol for quantifying β-hex release (Kuehn et al., 2010). In brief, bone marrow–derived mast cells seeded in a 96-well U-bottom plate were preincubated for 30 min at 37°C with either equal volumes of media alone, media and normalized plasma from vehicle-treated and tapered mice, or media and normalized plasma from DEX-treated and tapered mice; where appropriate, plasma concentrations first normalized to 300 ng/ml prior to addition. After washing, cells were incubated for 30 min at 37°C with 2 µg/ml DNP-IgE in media. Upon mast cell stimulation with 25 µg/ml DNP-HSA, β-hex released from mast cell granules was quantified using a colorimetric assay for digested *p*-nitrophenyl *N*-acetyl-β-D-glucosaminide substrate. The percentage of degranulated cells was calculated as β-hex content in the supernatant divided by total β-hex content in both the supernatant and the lysate combined.

### Online supplemental material

Fig. S1 shows that IgG1 switching is nearly unaffected by glucocorticoids. Fig. S2 shows glucocorticoids support the activation of IgE^+^ B cells and promote IgE production without global changes in chromatin accessibility. Fig. S3 shows glucocorticoid-inducible natural IgE may be locally produced in mLNs. Fig. S4 shows natural IgE is produced by local glucocorticoids in the gut, rather than by organismal stress conditions. Table S1 shows a list of defined vitamins, protein, and other components of B-27 supplement. Table S2 contains processed total RNA-seq data from B cells cultured ex vivo. Table S3 contains processed total RNA-seq data from sorted mLN B cells. Table S4 lists antibodies for flow cytometry. Table S5 lists primers for RT-qPCR.

## Supplementary Material

Table S1 lists defined vitamins, protein, and other components of B-27 supplement.

Table S2 shows total RNA-seq data from B cells cultured ex vivo.

Table S3 shows total RNA-seq data from sorted mLN B cells.

Table S4 lists antibodies for flow cytometry.

Table S5 lists primers for RT-qPCR.

SourceData F2 contains original blots for Fig. 2.
