# Supplementary material for: Induction of natural IgE by glucocorticoids
Source: J Exp Med. 2022 Sep 13;219(10):e20220903. doi: 10.1084/jem.20220903 (PMC9475297; doi:10.1084/jem.20220903)
Supplement: Table S1 — lists defined vitamins, protein, and other components of B-27 supplement. [file JEM_20220903_TableS1.docx]

### **Table S1. List of defined vitamins, protein, and other components of B-27 supplement**

###
