# Supplementary material for: Induction of natural IgE by glucocorticoids
Source: J Exp Med. 2022 Sep 13;219(10):e20220903. doi: 10.1084/jem.20220903 (PMC9475297; doi:10.1084/jem.20220903)
Supplement: Table S4 — lists antibodies for flow cytometry. [file JEM_20220903_TableS4.docx]

### **Table S4. List of antibodies for flow cytometry**

| **Target** | **Antibody** | **Company** | **Catalog number** | **Dilution** |
| --- | --- | --- | --- | --- |
| B220/CD45R | Monoclonal rat anti-mouse CD45R/B220 APC-eFluor 780 (clone RA3-6B2) | Invitrogen | 47-0452-82 | 1:200 |
|  | Monoclonal rat anti-mouse CD45R/B220 Brilliant UltraViolet 737 (clone RA3-6B2) | BD Horizon | 612839 | 1:200 or 1:400 |
|  | Monoclonal rat anti-mouse CD45R/B220 PE-Cyanine7 (clone RA3-6B2) | Invitrogen | 25-0452-82 | 1:200 |
| CD3 | Monoclonal rat anti-mouse/human CD3 Alexa Fluor 700 (clone 17A2) | Invitrogen | 56-0032-82 | 1:400 |
| CD5 | Monoclonal rat anti-mouse CD5 APC (clone 53-7.3) | BioLegend | 100626 | 1:200 |
| CD19 | Monoclonal rat anti-mouse CD19 APC-eFluor 780 (clone eBio1D3 (1D3)) | eBioscience | 47-0193-82 | 1:200 or 1:400 |
|  | Monoclonal rat anti-mouse CD19 PerCP-Cyanine5.5 (clone eBio1D3 (1D3)) | Invitrogen | 45-0193-82 | 1:200 or 1:400 |
| CD11b | Monoclonal rat anti-mouse CD11b Brilliant UltraViolet 737 (clone M1/70) | BD Horizon | 612800 | 1:200 |
|  | Monoclonal rat anti-mouse CD11b Brilliant Violet 711 (clone M1/70) | BD Horizon | 563168 | 1:200 |
| CD138/Syndecan-1 | Monoclonal rat anti-mouse CD138 APC (clone 281-2) | BioLegend | 142506 | 1:200 |
| CD16/32 | Anti-CD16/32 Fc block | eBioscience | 14-0161-86 | 1:100 or 1:200 or 1:400 |
| CD23/FcεRII | Monoclonal rat anti-mouse CD23 PE-Cyanine7 (clone B3B4) | Invitrogen | 25-0232-82 | 1:200 or 1:400 |
| CD38 | Monoclonal rat anti-mouse CD38 APC (clone 90) | eBioscience | 17-0381-82 | 1:400 |
| CD45 | Monoclonal rat anti-mouse CD45 Brilliant Violet 711 (clone 30-F11) | BioLegend | 103147 | 1:200 or 1:400 |
|  | Monoclonal rat anti-mouse CD45 Brilliant UltraViolet 395 (clone 30-F11) | BD Horizon | 564279 | 1:400 or 1:500 |
| CD93 | Monoclonal rat anti-mouse CD93 PE (clone AA4.1) | ThermoFisher | 12-5892-81 | 1:200 or 1:400 |
|  | Monoclonal rat anti-mouse CD93 PerCP-Cyanine5.5 (clone AA4.1) | Invitrogen | 45-5892-82 | 1:200 |
| CD95/Fas | Monoclonal Armenian hamster anti-mouse CD95 Brilliant Violet 510 (clone Jo2) | BD Horizon | 563646 | 1:200 |
| CD117/c-Kit | Monoclonal rat anti-mouse CD117/c-Kit APC (clone 2B8) | Invitrogen | 17-1171-82 | 1:200 or 1:400 |
|  | Monoclonal rat anti-mouse CD117/c-Kit APC/Cyanine7 (clone 2B8) | BioLegend | 105826 | 1:200 |
| FcεRI | Monoclonal American hamster anti-mouse FcεRI FITC (clone MAR-1) | eBioscience | 11-5898-82 | 1:200 or 1:400 |
|  | Monoclonal Armenian hamster anti-mouse FcεRI eFluor 450 (clone MAR-1) | eBioscience | 48-5898-82 | 1:200 |
| GR/NR3C1 | Unconjugated monoclonal rabbit anti-mouse/human glucocorticoid receptor (clone D6H2L) | Cell Signaling | 12041 | 1:200 |
| Live/dead | Zombie Red Fixable Viability Kit | BioLegend | 423110 | 1:400 |
|  | Zombie Yellow Fixable Viability Kit | BioLegend | 423103 | 1:200 or 1:400 |
| IgD | Monoclonal rat anti-mouse IgD Alexa Fluor 700 (clone 11-26c.2a) | BioLegend | 405730 | 1:200 or 1:400 |
|  | Monoclonal mouse anti-mouse IgD FITC (clone AMS 9.1) | BD Biosciences | 553507 | 1:200 |
| IgE | Monoclonal rat anti-mouse IgE Brilliant Violet 421 (clone R35-72) | BD Horizon | 564207 | 1:200 or 1:400 |
|  | Polyclonal goat anti-mouse IgE PE | Southern Biotech | 1110-09 | 1:200 or 1:400 |
| IgG (H+L) | Polyclonal goat anti-rabbit IgG (H+L) Alexa Fluor 488 | Invitrogen | A-11034 | 1:500 |
| IgG1 | Monoclonal rat anti-mouse IgG1 APC (clone X56) | BD Pharmingen | 550874 | 1:400 |
|  | Monoclonal rat anti-mouse IgG1 FITC (clone A85-1) | BD Pharmingen | 553443 | 1:200 |
| IgM | Monoclonal rat anti-mouse IgM Brilliant UltraViolet 395 (clone II/41) | BD OptiBuild | 743329 | 1:200 or 1:400 |
|  | Monoclonal rat anti-mouse IgM eFluor 450 (clone eB121-15F9) | Invitrogen | 48-5890-82 | 1:200 |
