# Supplementary material for: Induction of natural IgE by glucocorticoids
Source: J Exp Med. 2022 Sep 13;219(10):e20220903. doi: 10.1084/jem.20220903 (PMC9475297; doi:10.1084/jem.20220903)
Supplement: Table S5 — lists primers for RT-qPCR. [file JEM_20220903_TableS5.docx]

### **Table S5. List of primers for RT-qPCR**

| **Primer** | **Sequence (5′ to 3′)** |
| --- | --- |
| Ighe_F | GTTCGACCTGTCAACATCACT |
| Ighe_R | TAGAGGCTAGTTTGCCTTCCT |
| Ighg1_F | TGTGATCACCCAATCCCTGC |
| Ighg1_R | CCGGCACACAACAAAGAAGG |
| Ighm_F | GTGCCATTGGGATGGCTTTC |
| Ighm_R | ATCTTCCTCAGCAAGTCCGC |
| eGLT_F | AACCCTTTTGCTCAGGGTCC |
| eGLT_R | GAGATTCACAACGCCTGGGA |
| g1GLT_F | CAGGATCAATCCCAGCATTGGG |
| g1GLT_R | CTGTGCTTGGATCACCACACTTCC |
| mGLT_F | CTCTGGCCCTGCTTATTGTTG |
| mGLT_R | GAAGACATTTGGGAAGGACTGACT |
| Rpl13a_F | GAGGTCGGGTGGAAGTACCA |
| Rpl13a_R | TGCATCTTGGCCTTTTCCTT |
| Actb_F | GGCTGTATTCCCCTCCATCG |
| Actb_R | CCAGTTGGTAACAATGCCATGT |
| Cyp17a1_F | AGTGCTCGTGAAGAAGGGGA |
| Cyp17a1_R | TTTCCTTGGTCCGACAAGAGG |
| Hsd3b2_F | GGTTTTTGGGGCAGAGGATCA |
| Hsd3b2_R | GGTACTGGGTGTCAAGAATGTCT |
| Nr5a2_F | TGTGTGGCGATAAAGTGTCTG |
| Nr5a2_R | TCGACAGTAGGGACATCGTTT |
