# Supplementary figures and images for: Induction of natural IgE by glucocorticoids
Source: J Exp Med. 2022 Sep 13;219(10):e20220903. doi: 10.1084/jem.20220903 (PMC9475297; doi:10.1084/jem.20220903)

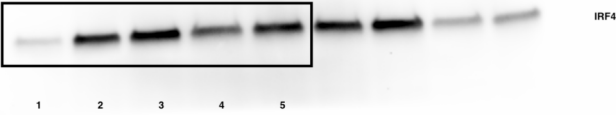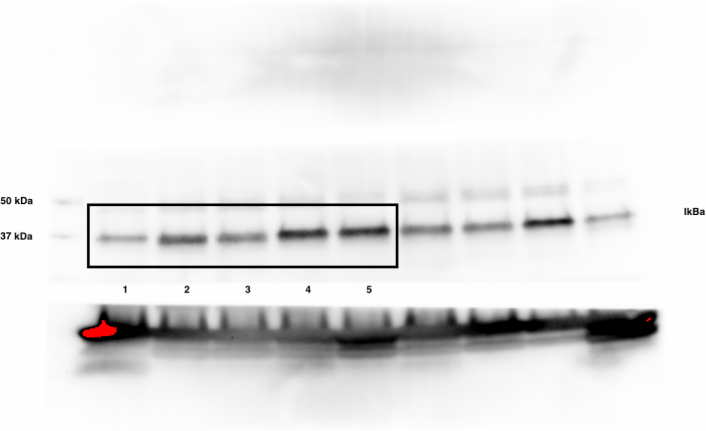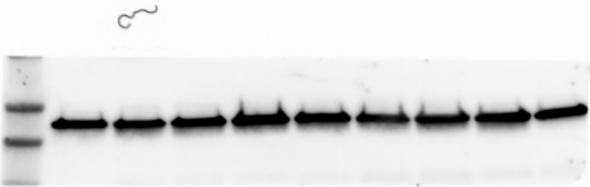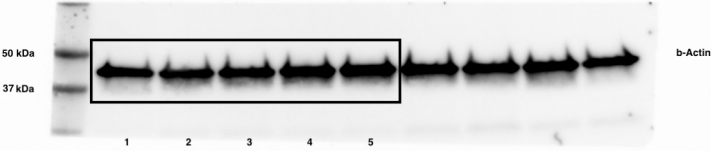

Supplement: SourceData F2 — contains original blots for Fig. 2. [file JEM_20220903_SourceDataF2.pdf]
